# Supplementary material for: Argipressin for prevention of blood loss during liver resection: a study protocol for a randomised, placebo-controlled, double-blinded trial (ARG-01)
Source: BMJ Open. 2023 Aug 24;13(8):e073270. doi: 10.1136/bmjopen-2023-073270 (PMC10450082; doi:10.1136/bmjopen-2023-073270)
Supplement: Supplementary data [file bmjopen-2023-073270supp001.pdf]

Clinical Study Protocol

Study product:	Argipressin
Study code:	ARG-01
EudraCT No:	2021-001806-32
Date:	230227

Influence of Argipressin on blood loss during liver resection; a double-blinded, randomized, placebo-controlled trial (ARG-01)

**Principle Investigator:**	Kristina Svennerholm, MD, PhD<br>Department of Anesthesia and Intensive Care<br>Sahlgrenska University Hospital<br>413 45 Gothenburg, Sweden	Tel: +46 31 3429336<br>kristina.svennerholm@vgregion.se
**Study centre:**	Department of Anesthesia and Intensive Care<br>Sahlgrenska University Hospital<br>413 45 Gothenburg, Sweden	Tel: +46 31 3428607<br>caroline.pihl@vgregion.se<br>(Department Assistant)
**Co-investigator:**	Ellinor Wisén, MD<br>Department of Anesthesia and Intensive Care<br>Sahlgrenska University Hospital<br>413 45 Gothenburg, Sweden	Tel: +46 31 3428171<br>ellinor.wisen@vgregion.se
**Co-workers:**	Lena Sand-Bown, MD, PhD<br>Department of Anesthesia and Intensive Care<br>Sahlgrenska University Hospital<br>413 45 Gothenburg, Sweden	Tel: +46 31 3428157<br>lena.sand@vgregion.se
	Andreas Kvarnström, MD, PhD<br>Department of Anesthesia and Intensive Care<br>Sahlgrenska University Hospital<br>413 45 Gothenburg, Sweden	Tel: +46 31 3426617<br>andreas.kvarnstrom@vgregion.se
	Sven-Erik Ricksten, MD; PhD, Professor<br>Department of Anesthesia and Intensive Care<br>Sahlgrenska University Hospital<br>413 45 Gothenburg, Sweden	Tel: +46 31 3427433<br>sven.erik.ricksten@vgregion.se

Magnus Rizell, MD, PhD
Department of Transplantation Surgery
Sahlgrenska University Hospital
413 45 Gothenburg, Sweden
Tel: +46 70 5259301
magnus.rizell@vgregion.se

Study monitor: Gothia Forum
Sahlgrenska University Hospital
413 45 Gothenburg, Sweden
Tel: +46 31 3429679

Sponsor: Region Västra Götaland,
Sahlgrenska University Hospital
Kristina Svennerholm,
Department of Anaesthesia and Intensive Care,
Sahlgrenska University Hospital
413 45 Gothenburg, Sweden

The following amendments and administrative changes have been made since final protocol date:	
original	Oct 26 2021
Version 2	Dec 2, 2021<br>Clarifications regarding access to data (2.3), pregnancy at inclusion (5.2), procurement and handling of the study drug (6.1.2), randomization process (6.1.3), statistic methods (10).)
Version 3	Feb 27, 2023<br>The title is updated. Clarifications regarding collection of samples, access to data and data entry into the eCRF and planned publication of results. Figure 1 is updated and clarified. The entire statistics chapter (9) is updated. WHO trial registration data set and Statistical analysis plan is supplemented. Changes from version 2 are marked in yellow.

Supplements:

1. World Health Organization Trial Registration Data Set
2. Statistical Analysis Plan
3. Patient information

Appendixes:

Appendix A: Signatures
Appendix B: Ethics and regulatory review
Appendix C : Definition of adverse events
Appendix D: Label

Synopsis

Title: Influence of Argipressin on blood loss during liver resection; a double-blinded, randomized placebo-controlled trial (ARG-01).

Principle Investigator
Kristina Svennerholm, MD, PhD, Dpt of Anesthesia and Intensive Care, Sahlgrenska University Hospital

Study centre
Sahlgrenska University Hospital and University Hospital of Northern Sweden (NUS)

Study period
First patient in: March 2022, last patient out December 2024

Short background
Hepatic resection is a major surgical intervention with high risk of substantial blood loss. The surgical means to reduce blood loss may impair perfusion and induce intestinal congestion. If blood flow to the liver can be influenced by pharmacological means, blood loss and transfusion requirements may be reduced. Moreover, the inflammatory system is involved in cancer development, and the anti-inflammatory properties of Argipressin may decrease the inflammatory response after hepatic surgery.
Even though several studies have been made on Argipressin and other pharmacological treatments during hepatic surgery, Argipressin has not yet been proven to reduce blood loss in patients treated according to our protocol, and the effects of Argipressin on the inflammatory response in this setting is not explored.

Hypothesis
Infusion of Argipressin during hepatic resection surgery reduces blood loss. It may reduce transfusion requirements, and mitigate the perioperative inflammatory response compared to placebo.

Name and description of study product
Argipressin (Empressin®) is an endogenous substance, and part of the body's response to stress and trauma. Argipressin affects V1-receptors to produce vasoconstriction, and V2-receptors in the kidney to enhance water retention. It is also involved in inflammatory reactions, and affects platelets.

Primary objective
To determine if infusion of Argipressin during hepatic resection surgery reduces blood loss compared to placebo.

Secondary objectives
-To determine if infusion of Argipressin during hepatic resection surgery reduces the need of transfusions during and after surgery, compared to placebo.
-To determine if infusion of Argipressin during hepatic resection surgery mitigates the postoperative inflammatory response, compared to placebo.
-Analyse effects of infusion of Argipressin during hepatic resection surgery on biomarkers of renal, cardiac and intestinal damage, perioperative data and hemodynamic measurements.

Primary endpoint:
Blood loss (ml) at the end of surgery.

Secondary endpoints:
Volume of transfused blood during surgery and postoperative day 1, 2 and 5, respectively. Inflammatory markers during and after surgery. Markers of kidney, cardiac and intestinal damage will be analysed, and perioperative data as well as 30 day follow up data will be recorded.

Study design:
A single center, double blinded, randomized, parallel group, placebo- controlled trial to investigate the effect of Argipressin on blood loss, transfusion requirements and inflammatory response during hepatic surgery.

Inclusion criteria
-Participant planned for hepatic resection (open or laparoscopic, regardless of indication for surgery).
-Age ≥18 years.
-American Society of Anaesthesiologists (ASA) class I-III.
-Signed informed consent form.

Exclusion criteria
-Participant does not understand the given information, and/ or cannot give written informed consent.
-Simultaneous operation of tumor with other localization, or surgery for superficial single hepatic tumor less than 2 cm, expected to be of short duration and with minimal blood loss.
-Terminal kidney failure (estimated preoperative GFR< 15 ml/min)
-Pregnancy or lactation.
-Known allergy to Empressin®.
-Patient included in other interventional study, interacting with the endpoints in the present study, or previous randomization in this study.
-Hyponatremia (S-Na < 130 mmol/L)
-Patient considered ineligible for other surgical or medical reason.
-Present infection.
Patients with systemic inflammatory disease, inflammatory bowel disease or preoperative corticosteroid treatment will not be eligible for the subgroups where cytokines and interleukins are investigated.

Study medicine/product
Intravenous infusion of Empressin® 0.8 U/ml, 0.045 U/kg/h (0.056 ml/kg/h). Infusion is started after induction of anesthesia, and stopped at the end of surgery. NaCl 9 mg/ml (“normal saline”) given with the same infusion rate will be used as placebo.

Statistics
Data will be analysed using linear regression on log-transformed data. Correlation with perioperative variables will be explored retrospectively. Demographic, anaesthesiologic and surgical data, as well as hemodynamic recordings, will be presented as descriptive data. Calculation of sample size was performed based on data from open and laparoscopic hepatic resections performed at Sahlgrenska University Hospital during the period January 2019- October 2020. Adverse Events will be registered. Serious Adverse Events will be reported.

Table of contents

	Synopsis	4
	Abbreviations	8
1	Background	10
1.1	Rational for conducting this study	11
1.2	Hypothesis	11
1.3	Risk/benefit evaluation	12
1.4	Ethics and Regulatory review	13
2	Objectives and Endpoints	14
2.1	Primary objective	14
2.2	Secondary objectives	14
2.3	Primary endpoint	14
2.4	Secondary endpoints	14
3	Study Design	15
3.1	Overall study design	15
3.1.1	Stopping criteria	18
3.1.2	Rational for study design, dose and laboratory analysis	18
4	Patient Population	19
4.1	Inclusion criteria	19
4.2	Exclusion criteria	19
4.3	Subject enrolment	20
4.4	Discontinuation and withdrawal of subjects	20
4.5	Premature termination of the stud	21
5	Methodology	21
5.1	Treatment	21
5.1.1	Labelling	22
5.1.2	Storage, handling and delivery	22
5.1.3	Randomization, Blinding and Study Drug administration	23
5.1.4	Treatment compliance	23
5.1.5	Accountability	23
5.2	Samples and Biobank	24
5.3	Concomitant medication	24
6	Study measurements	25
6.1	Measurements, time points and procedures	25
6.1.1	Preoperative visit	25
6.1.2	Day of surgery	25
6.1.3	Postoperative procedures	27
6.1.4	Follow up	27
6.2	Recording of data	28
6.3	Study variables and laboratory analysis	28
6.3.1	Study variables	28
6.3.2	Blood and urine	28
6.3.3	Safety measurements and variables	29

7	Safety	30
7.1	Expected adverse reactions	30
7.2	Definitions of Adverse Events (AE)	31
7.2.1	Definition of severity rating	31
7.2.2	Causal relationship	31
7.3	Definitions of Serious Adverse Events	32
7.3.1	Recording and reporting of Adverse Events	32
7.3.2	Follow-up of unresolved Adverse Events	32
7.3.3	Suspected Unexpected Serious Adverse Reaction	32
7.3.4	Reporting of Suspected Unexpected Serious Adverse Reaction	33
7.3.5	Reference safety information	33
8	Study Management	33
8.1	Data Management	33
8.2	Monitoring	33
8.3	Archiving	34
8.4	Quality assurance	34
8.4.1	Audits and Inspections	34
8.4.2	Significant changes	35
8.5	Insurances	35
9	Statistics	35
10	Report and publication	37
11	Study timetable	37
11.1	Definition of “End of study”	37
12	Declaration of interests and funding	38
13	References	39

Abbreviations

AE	Adverse event
AKI	Acute Kidney Injury
Alb	Albumin
ALT	Alanine Transaminase
AST	Aspartate Transaminase
ASA-class	American Society of Anaesthesiologists classification
AVP	Arginine Vasopressin
C3a	Complement factor 3a
C5b-9	Terminal Complement Complex 5b-9
CFS	Clinical Frailty Scale
CVP	Central Venous Pressure
CRP	C-Reactive Protein
eCRF	Electronic Case Report Form
EC	Ethics Committee, synonymous to Institutional Review Board and Independent Ethics Committee
ECG	Electrocardiogram
GCP	Good Clinical Practice
GMP	Good Manufacturing Practice
HABR	Hepatic Artery Buffer Response
Hb	Haemoglobin
hs-TNI	high sensitive -Troponin-I
ICH	International Council for Harmonisation of Technical Requirements for Pharmaceuticals for Human Use
ICAM-1	Intercellular Adhesion Molecule -1
IFABP	Intestinal Fatty Acid Binding Protein
IGFBP7	Insulin-like growth factor-binding protein 7
IL-1β IL-6, IL-8, IL-10	Interleukin -1β, -6, -8, -10.
IMP	Investigational medical product
ITT	Intention To Treat
MCP-1	Monocyte Chemoattractant Protein -1
PT-INR	Prothrombine Time- International Normalized Ratio
PLT	Platelet Count
SAE	Serious Adverse Events
SAP	Statistic Analysis Plan
SDF-1	Stromal Cell Derived Factor -1
SMPA	Swedish Medical Products Agency
SUSAR	Suspected Unexpected Serious Adverse Reaction
TIMP-2	Tissue Inhibitor of Metalloproteinase 2
TRALI	Transfusion Induced Lung Injury

WBC	White Blood Cell Count

Background

Hepatic resection due to colo-rectal cancer metastasis, primary hepatic or gallbladder cancer, or benign conditions, is a major surgical intervention. The surgery entails a high risk of perioperative circulatory instability and substantial blood loss. Blood loss and blood transfusion is associated with morbidity and mortality after liver surgical treatment of malignant disease (1, 2, 3). Standard anaesthesiologic management of these patients aim to minimize blood loss by reducing CVP, and to the same end surgical treatment includes mechanical occlusion of the hepatic circulation (Pringle manoeuvre) (4).
Argipressin (also known as Arginin-Vasopressin, AVP or Vasopressin) is an endogenous substance, produced in the hypothalamus and released from the posterior pituitary gland as part of the body's response to stress and trauma. Among other effects, Argipressin induces vasoconstriction by V1 receptors in smooth muscle (prominent in the splanchnic circulation), and water retention by V2 receptors in the kidney's collecting ducts. Argipressin and its analogues are established agents for treatment of vasoplegia, for example in sepsis where endogenous levels are low, as well as treatment of esophageal varices and hepatorenal syndrome. It may be used during anesthesia for liver transplantation, and our group has shown that it decreases portal and hepatic blood flow in patients undergoing hepatic resection (5, 6).

Pringle's manoeuvre, or surgical occlusion of the hepatoduodenal ligament, is used during hepatic surgery to limit the blood supply to the liver to minimize blood loss. This method impairs venous drainage from mesenteric vessels, and induce stasis of the bowel and intermittent hepatic ischemia/ hypoperfusion as well as frequent circulatory instability and lactate production (7). When the blood flow through the portal vein is reduced, the flow is reciprocally increased in the hepatic artery (the Hepatic Artery Buffer Response, HABR) (8). Argipressin may redirect blood flow from the splanchnic circulation, and hence decrease blood flow through the liver while maintaining oxygenation of the liver parenchyma by means of the HABR. This redirection of blood flow may be a way of reducing blood loss during hepatic resection. Abbas et al showed a significant reduction of blood loss with the use of Terlipressin (an Argipressin-analogoue, with comparatively longer half-life) during open hepatic resection, but they did not evaluate effects on inflammatory response or on organ perfusion (9). Sims et al administered an Argipressin bolus followed by infusion for 48 hours to trauma patients with ongoing hemorrhage, and noted decreased transfusion requirements in the treatment group compared to placebo (10).

Major surgery such as hepatic resection triggers the inflammatory response (11). The inflammatory system has a key function in cancer development, and Argipressin may have anti-inflammatory properties (12, 13).

As for any vasoactive agent, the vasoconstrictive properties of Argipressin imply a risk of reduced perfusion of internal organs, such as kidneys and intestines, and there are

conflicting results in this area of research. Some studies suggest improved kidney function after use of Argipressin analogues during liver transplantation, while others show an oxygen supply-demand mismatch in the kidney when used adjacent to cardiac surgery (14, 15, 16). The large randomized controlled studies VASST and VANISH on sepsis patients did not demonstrate any increased risk of acute kidney injury for the patients treated with Argipressin, and the VANISH study showed a reduced need for renal replacement therapy (17, 18). The effect of Argipressin on the mesenteric circulation may be beneficial, as when used to reduce blood flow to treat esophageal varices. On the other hand, the reduced flow might also imply a risk of intestinal hypoperfusion. The same line of reasoning applies to the cardiac circulation (19).

1.1 Rational for conducting this study

The most common indication for hepatic resection is colo-rectal cancer metastasis, but other conditions such as tumours in the biliary system, primary hepatic tumours and benign tumours may require surgical treatment. Colo-rectal cancer is a common malignant disease, and a large number of patients are affected by the outcome of surgical treatment. Blood loss and blood transfusion is shown to influence outcome negatively for patients undergoing surgical treatment, and transfusion of blood products may initiate immunological reactions (1, 2, 3). The surgical means to reduce blood loss during hepatic surgery may impair perfusion and induce intestinal congestion. If blood flow to the liver can be influenced by pharmacological means, thus reducing blood flow to the liver while maintaining oxygenation through the HABR, blood loss and transfusion requirements may be reduced. Ultimately, this could influence morbidity and mortality. The inflammatory system is involved in cancer development and is triggered after major surgery. Anti-inflammatory properties of Vasopressin may decrease the inflammatory response after hepatic surgery and enhance recovery.

The intention of this study is to increase the knowledge of Argipressin to the benefit of future patients. Abbas et al studied Terlipressin effects on hepatic surgery patients, albeit with a more generous fluid therapy approach compared to the low CVP technique used in our centre and showed a significant reduction of blood loss (9). The median blood loss for major open resections in our centre was 1250 ml during the period January 2019- October 2020, and effects of Argipressin has not yet been investigated in the setting of our relatively fluid restrictive protocol, with an increasing number of laparoscopic hepatic resections. We also want to investigate the potential anti-inflammatory effects of Argipressin in this setting.

1.2 Hypothesis

Infusion of Argipressin during hepatic resection reduces blood loss during hepatic surgery. It may also reduce transfusion requirements, and mitigate the perioperative inflammatory response compared to placebo.

1.3 Risk/benefit evaluation

Blood loss and transfusion is shown to influence outcome negatively after surgery for colo-rectal metastases. Inflammatory reactions are expected after major surgery, and mitigation of this response may improve recovery. By potentially influencing these factors, treatment with Argipressin may be beneficial for the individual patient, as well as reducing health care needs for this large group of patients. Reduced needs of donated blood products would also be beneficial in a socioeconomic perspective.

Argipressin is an established pharmacological agent in anaesthesia and intensive care for treatment of vasoplegia and circulatory instability, together with norepinephrine or as a single agent. Several studies show beneficial effects on kidney function after use of Argipressin, for example after liver transplant surgery, and it is recommended in sepsis treatment guidelines (5, 14, 20, 21). Sims et al investigated the effects of Argipressin on transfusion requirements in trauma patients with severe haemorrhage, and showed reduced need of transfusion in the treatment group without any difference in mortality, morbidity or complications (10).

As all vasoconstrictive agents, Argipressin may impair organ perfusion, and in extreme cases intestinal or digital ischemia may arise. Patients will be closely monitored during the period of Argipressin infusion and during the postoperative period, with visual observation of signs of ischemia, and laboratory tests. In this study, the infusion of Argipressin will be given only during surgery, i. e. during a limited time of a few hours, compared to the VANISH, VASST and Sims' studies where the study drug was infused during several days (10, 17, 18). Patients will be euvolemic, and not in septic or haemorrhagic shock. The dose of Argipressin will be 0.045 U/kg/h (corresponding to a dose of 3.6 U/h for a patient of 80 kg), which is equal to the dose used in the VANISH-trial, and somewhat higher than the dose used in the VASST trial, but still lower than the maximal dose in other studies including previous studies at our centre (6, 22). The half-life of Argipressin is approximately 10 minutes, and hence no circulatory effects of the infusion will be present after the immediate postoperative period. The placebo infusion (NaCl 0.056 ml/kg/h) will not have any circulatory or pharmacologic effects.

All treatment except for the study drug will be according to clinical routine, and no extra visits, X-rays or clinical investigations will be required. The extra amount of blood drawn for blood samples in the study is approximately 70 ml, and compared to a normal total blood volume of approximately 5000 ml considered negligible in this context. Argipressin is not expected to interact with any treatment or medication used in clinical routine.

Data will be registered in Red Cap®, and will only be accessible to the study nurses, the investigators and the monitor before database lock. The study nurses and the investigator will not have access to the treatment codes until after database lock. All data will be

handled according to Swedish law. The study will not be associated with extra travels or visits, and the patient population is prioritized for cancer treatment regardless of the Covid -19 pandemic. The study will be subject for external monitoring, and reporting of AE:s will be performed according to Swedish regulations for clinical trials. The investigators will consider the EMA guidance, "Points to consider on implications of Coronavirus disease (COVID-19) on methodological aspects of ongoing clinical trials" (EMA/158330/2020 Rev. 1).

In summary we consider the potential risks associated with infusion of Argipressin during a limited time to be minimal, in relation to the inherent risks of major abdominal surgery, and corresponding doses of Argipressin have been used during several days in other studies.

1.4 Ethics and Regulatory review

The study will be conducted in compliance with the International Council for Harmonisation of Technical Requirements for Pharmaceuticals for Human Use (ICH), Good Clinical Practice (GCP), applicable national regulatory requirements (Swedish Medical Products Agency (SMPA) regulations 2011:19) and in accordance with the ethical principles in the Declaration of Helsinki (2013).

The Swedish Ethical Review Authority (Etikprövningsmyndigheten, EPM) has approved the study (DNR 2021-03557), and the study is registered at the ClinicalTrials.gov (NTC05293041) and the EudraCT databases (EudraCT 2021-001806-32).

The investigators will ensure that the subjects are given full and adequate oral and written information about the nature, purpose, possible risks and benefits of the study, and ensure that the subjects are notified that they are free to discontinue from the study at any time. Signed and dated informed consent will be obtained before any study specific procedures are conducted, and stored in the Study Master File. Each participant will receive a copy of the signed Informed Consent Form. The final version of the informed consent form and other information provided to subjects is approved by EPM. EPM and the Swedish Medical Products Agency will be informed of any changes in the study protocol in accordance with current requirements.

All data will be handled in accordance with the EU Directive 2016/679 (GDPR) and the Law (2018:218) with additional regulations on the EU data protection regulation. If any part of the data is handled by any other organization, inside or outside the EU, appropriate agreements and/or other documentation will be established, to ensure that the data processing is performed in accordance with the provisions of the General Data Protection Regulation (GDPR) and other relevant legislation, before any data transfer takes place.

For detailed information regarding ethics and regulatory review, informed consent, data protection and audits as well as inspections see **Appendix B**.

The Ethics review board and the SMPA have been informed about the updates of the protocol.

1. Objectives and Endpoints

2.1 Primary Objective

To determine if infusion of Argipressin during hepatic resection surgery reduces blood loss compared to placebo.

2.2 Secondary Objectives

- To determine if infusion of Argipressin during hepatic resection surgery reduces the need of transfusions during and after surgery, compared to placebo.
- To determine if infusion of Argipressin during hepatic resection surgery mitigates the postoperative inflammatory response, compared to placebo.

2.3 Primary Endpoint

Blood loss (ml) at the end of surgery, measured according to the investigator's instructions, by visual assessment of suction devices and gauze, and subtraction of ascites and irrigation fluids.

2.4 Secondary Endpoints

- Blood transfusion (ml) at the end of surgery, at postoperative day 1 and postoperative day 2 or 5, respectively.
- Levels of WBC, CRP, PLT and Albumin at the end of surgery and postoperative day 1-5.
- Levels of IL-1β IL-6, IL-8, IL-10, MCP-1, SDF-1α, ICAM, C3a, C5b-9 at the end of surgery and postoperative day 1 and 2.

To evaluate effects of the study drug on kidneys, heart and intestines, tissue biomarkers will be part of the study protocol:

- Plasma creatinine will be controlled at postoperative day 1, 2 and 5. Urine [TIMP-2] x [IGFBP-7] will be controlled after surgery.
- Hs-TNI will be controlled at the end of surgery and at postoperative day 1.
- Arterial lactate will be controlled at the end of surgery, at three hours after surgery and at postoperative day 1.
- IFABP will be controlled at three hours after surgery and postoperative day one.

Perioperative data and hemodynamic measurements as listed below will be recorded and presented as descriptive data.

- Vasopressor use during surgery, achievement of CVP goal, total urine output and use of furosemide at postoperative day 1.
- Measurements of mean arterial blood pressure (MAP), heart rate, CVP and Cardiac Index (if applicable), as well as assessment of capillary refill time in digits at the end of surgery.
- Total time of surgical hepatic occlusion and radicality of resection.
- Length of stay in hospital and postoperative complications documented at postoperative day 30.

3. Study Design

3.1 Overall study design

This single centre, double blinded, randomized placebo- controlled trial is performed at Sahlgrenska University Hospital. The aims are to investigate the effect of Argipressin on blood loss, transfusion requirements and inflammatory response during hepatic surgery. Participants will be stratified according to planned surgical technique (open or laparoscopic surgery) and planned extent of resection (“large” or ”small”) before randomization to treatment with argipressin or placebo during surgery. Markers of kidney, cardiac and intestinal damage will be evaluated during and after surgery. More extensive testing of inflammatory markers will be performed in a limited number of patients (subgroup) in each arm at Sahlgrenska University Hospital.

The study drug infusion will be started after induction of anesthesia and continue until the end of surgery. All other treatment will be according to clinical routine, including use of vasopressor (norepinephrine) if required to achieve adequate MAP. Blood loss will be assessed at completion of surgery, and given transfusions recorded at “End of surgery” and “postop day 1”. Blood and urine samples will be obtained before and during surgery (timepoints: Day of surgery; “Before anesthesia”, “After induction”, “End of surgery), as well as postoperatively (timepoints: “End of surgery +3h”, “Post op day 1-5”). Assessments at postoperative day 5 will pertain only to patients operated with open technique, since patients operated with laparoscopic technique are expected to be discharged from hospital on postoperative day 2. Perioperative data and hemodynamic measurements will be registered during surgery.

Throughout the study, the treating physician will be clinically responsible for the patient. This includes interpretation of laboratory results obtained during the hospital stay, treatment of surgical or medical conditions and consultation of other specialists if needed. No further measurements or samples will be obtained after the fifth postoperative day. Data from the surgical follow up at 30 days will be obtained from the SweLiv registry and recorded in the eCRF. Adverse reactions and adverse events are registered in the eCRF throughout the entire study, i. e. until postoperative day 5.

Overall study design and activities are shown in Figure 1 and Table 1 below.

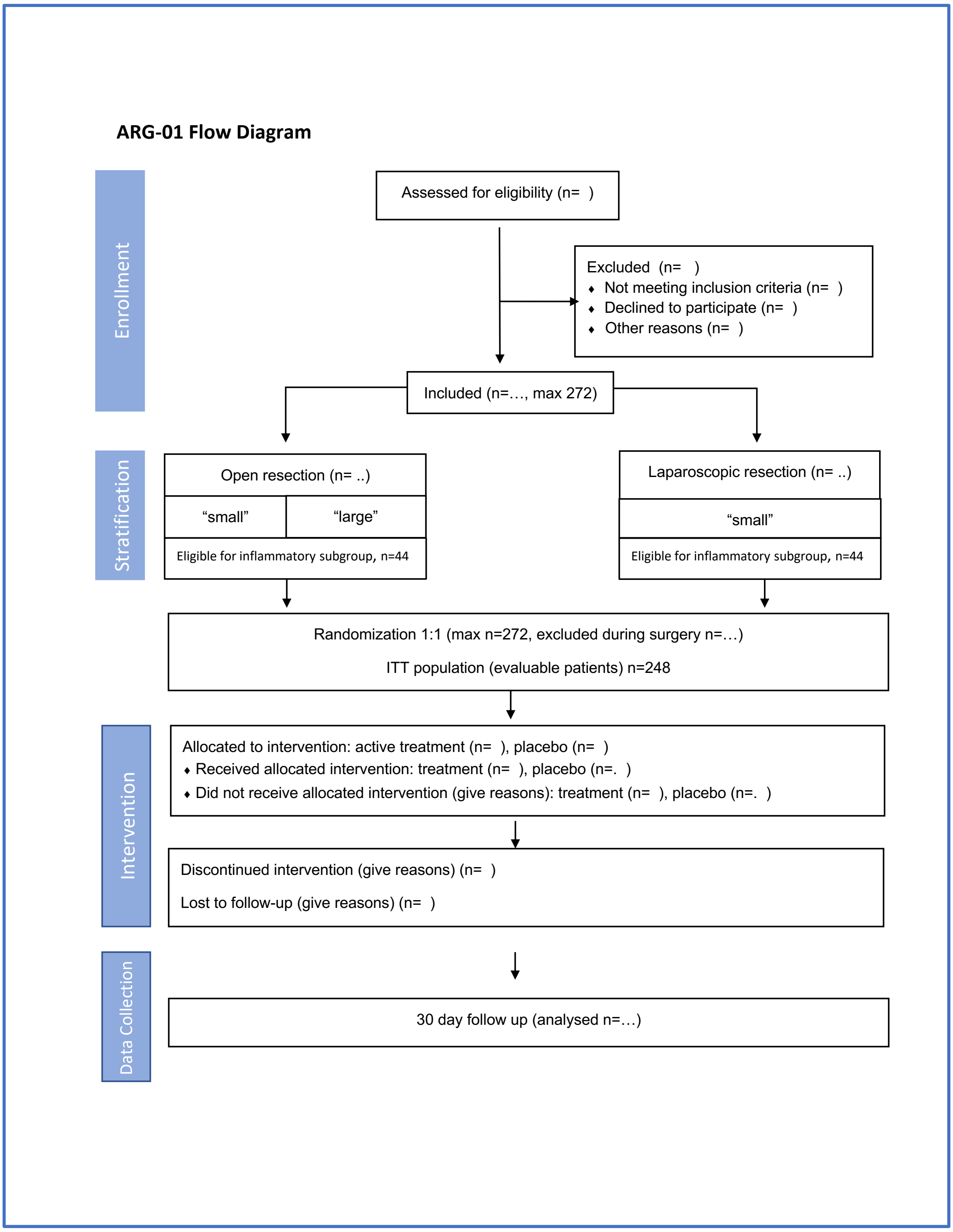

Fig 1. Flow Chart ARG-01.

Table 1 - Overview of the study activities

	Enrolment	**Allocation**	**Intervention**				**Post operative care →**				***Close -out***
**TIMEPOINT**	***Pre op visit***	**Day of surgery -** *Before anesthesia*	**Day of Surgery-** *After induction*	**Day of Surgery-** *End of surgery*	**Day of Surgery-** End of Surgery +3h	***Post op day 1*** *5 am*	***Post op day 1*** *10 am*	***Post op day 2***	***Post op day 5****	***30 day follow up***
**ENROLMENT:**										
Eligibility screen	X									
Informed consent	X									
Demographic data	X									
Allocation		X								
**INTERVENTION:**										
Study drug			I--------------	-----------I						
assessment of AEs		X	X	X	X	X	X	X	X	
**ASSESSMENTS:**										
Blood loss				X						
Blood transfusion (ml)				X			X	X	X	
Norepinephrine**				X						
Use of other inotropic drugs				X						
CVP-goal achieved				X						
Total diuresis							X			
Furosemide total dose							X			
Hemodynamics^			X	X						
Surgical data^^				X						
Postoperative data^^^										X
Clinical Frailty Scale	X									
**LABORATORY TESTS:**										
Blood samples•		X		X		X		X	X	
Cytokines +Interleukins••		X		X			X	X		
[TIMP-2 xIGFBP7], U-creatinine			X		X					
hs-TNI		X		X		X				
Arterial lactate, glc, Na	X°	X		X	X	X				
IFABP		X			X		X			
AST, ALT, PT-INR	X					X		X	X	

*Patients having open surgery, 4-6 days postoperative, closest possible weekday;
**Total dose of norepinephrine during surgery (mcg), divided by minutes of surgery and weight of patient;
^Mean Arterial Blood Pressure, Heart Rate (before induction), Central Venous Pressure and Cardiac Index (after insertion) and thereafter every 30 min during the resection phase. Capillary refill time; hourly during surgery
^^Performed surgical procedure, Duration of surgery, total Pringle time
^^^complications (SweLiv Registry), length of stay in hospital, radicality of resection
•WBC, CRP, PLT, Albumin, Hemoglobin, p-Creatinine
••cytokines / interleukins: IL-1β IL-6, IL-8, IL-10, MCP-1, SDF-1α, ICAM, C3a, C5b-9.
°only S-Na

3.1.1 Stopping criteria

The only general stopping criteria is suspected hypersensitivity to the study drug or to any of the excipients. In case of unexpected serious events jeopardizing the safety of the subject, the study may be stopped by the physician in charge, and the randomization code broken. Any data recorded in Red Cap® will be kept, but no further recordings will be made. The time of and reason for study drug termination will be recorded. Any saved blood samples collected until then will be discarded, but data already entered into the eCRF will be included in the study analysis.

3.1.2 Rational for study design, dose and laboratory analyses

Design

Participants will be stratified according to planned surgical technique and planned size of resection, since open and larger resections have a higher expected blood loss. Randomization will be performed by a computerized randomization tool in Red Cap®, at a 1:1 ratio. From each allocation arm, 44 (total n=88) patients will be selected for extended testing of cytokines and interleukins. These tests are costly and require immediate centrifugation and freezing and will therefore not be performed in all the study participants.

Study drug infusion and dose

The study drug infusion will be started after induction of anaesthesia and collection of baseline blood and -urine samples. The study drug infusion (Empressin® 0.8 U/ml or NaCl 9mg/ml (placebo)) is run at a constant speed of 0.056 ml/kg/h until the end of surgery. This corresponds to a dose of Empressin® of 0.045 U/kg/h, or 3.6 U/h for an 80 kg patient (Empressin® 0.8 U/ml, 0.056 ml/kg/h= 4.5 ml/h = 3.6 U/h for 80 kg). Empressin® has a half-life of 10 minutes in the circulation, and steady state in plasma is reached after 30 minutes of infusion. Starting the infusion at the beginning of surgery will give the infusion time to reach steady state and potentially divert blood away from the mesenteric circulation before start of the resection of liver parenchyma.

The dose of Argipressin (Vasopressin) in previous large RCTs varies between 0.03- 0.075 U/kg/h (10, 17, 18). In the Surviving Sepsis Campaign guidelines, the recommended dose is 0.03 U/min (1.8 U/h), which is also suggested in FASS, in both cases for use during 48 hours (20). The dose of Argipressin used in this study is well established in our hospital clinical practice, and lower than the dose used in previous studies at our center (6, 22).

Choice of laboratory analyses

Inflammatory markers are expected to rise after major surgery. CRP, WBC, PLT and Albumin are controlled postoperatively according to clinical routine, and are also part of the study protocol outcome measurements for all included subjects. The analyses of interleukins and cytokines (IL-1β IL-6, IL-8, IL-10, MCP-1, SDF-1α, ICAM, C3a, C5b-9) were chosen according to our pilot study to represent different stages of the inflammatory cascade and is performed in a subgroup of 44 patients in each arm.

Plasma creatinine is controlled in clinical practice as a marker of kidney dysfunction, and is used in the definition of AKI according to the KDIGO criteria. Urine [TIMP-2] x [IGFBP-7] (Nephrocheck®) is suggested (although not validated) as a marker of early tubular damage,

and urine-creatinine will be analysed at the same timepoints to enable comparison of Nephrocheck® values.

hs-TNI is a specific marker of cardiac ischemia. Hs-TNI is followed until post op day 1; any increase after that will not likely be due to the study drug infusion. In case hs-TNI is not available (not provided at all centres), TNT will be analysed and presented separately.

Arterial lactate will be controlled frequently according to clinical practice, together with plasma glucose levels (since the glucose level itself might influence lactate values). IFABP is suggested as a marker of intestinal mucosal damage (23). In our pilot study, IFABP returned to baseline at Postoperative day 1, and will not be followed after that timepoint. Serum-Na will be controlled due to Argipressin's potential to cause water retention.

AST, ALT, PT-INR and Haemoglobin are controlled according to clinical practice. The results will be recorded in the eCRF and presented as descriptive data.

The measurement timepoints were chosen to cover the peak increase of the various laboratory tests. Some of the measurements are also considered safety parameters (plasma creatinine, hs-TNI, arterial lactate).

4. Patient Population

All patients > 18 years, male and female, scheduled for hepatic resection at Sahlgrenska University Hospital due to primary malignant disease, metastasis or benign tumour will be assessed for eligibility. The surgery schedule is reviewed together with study nurses and surgical coordinator to make sure all eligible patients are considered.

In each arm (treatment/ placebo, stratified for open or laparoscopic resection), 44 patients (total n= 88) will be selected for extended testing of inflammatory markers. This selection will be based inflammatory status and logistic factors. The patient will be marked as eligible for the inflammatory subgroup in the randomisation part of Red Cap®, to ensure adequate distribution of the subgroup patients.

Participants will have to fulfil the following inclusion and exclusion criteria:

4.1 Inclusion criteria

1. Participant planned for hepatic resection (open or laparoscopic, regardless of indication for surgery).
2. Age ≥18 years.
3. ASA class I-III.
4. Signed informed consent form.

4.2 Exclusion criteria

1. Participant does not understand the given information, and/ or cannot give written informed consent.

2. Simultaneous operation of tumor with other localization, or surgery for superficial single hepatic tumor less than 2 cm, expected to be of short duration and with minimal blood loss.
3. Terminal kidney failure (estimated preoperative GFR< 15 ml/min)
4. Pregnancy or lactation (Patients declare any possibility of pregnancy at the preoperative assessment, and if appropriate a pregnancy test will be taken).
5. Known allergy to Empressin®.
6. Patient included in other interventional study, interacting with the endpoints in the present study, or previous randomization in this study.
7. Hyponatremia (S-Na < 130 mmol/L)
8. Patient considered ineligible for other surgical or medical reason.
9. Present infection (defined as active treatment with antibiotics, prophylactic antibiotics is not an exclusion criteria).

Patients with systemic inflammatory disease, inflammatory bowel disease or preoperative corticosteroid treatment will not be eligible for the subgroups where cytokines and interleukins are investigated.

4.3 Subject enrolment

The investigator will keep a screening list of patients who were considered for enrolment but not included, and record the reason for exclusion. The identity of the patients on the list will not be available to the rest of the research group. Immediately after inclusion of a participant fulfilling the inclusion/exclusion criteria, a Signed Informed Consent will be obtained before any study-specific procedures are carried out. Information is given, and consent form obtained by the study staff physicians or specifically trained physicians at the preoperative anaesthesia consultation department. Furthermore, a “subject number” will be allocated to each subject. The subject number will follow on all patient records, eCRFs, urine- and blood samples etc, except for blood samples sent directly to the department of Clinical Chemistry for analysis. Those samples will be labelled with the patient’s social security number, and recorded in the hospital clinical charts. The results will then be transferred to the eCRF under the subject number.

4.4 Discontinuation and withdrawal of subjects

Subjects are free to discontinue their participation in the study at any time without prejudice to further treatment. If participation is discontinued, subjects will be asked about the reason(s) for the discontinuation and the presence of any AE. The subjects may be withdrawn from the study at the discretion of the investigators due to safety concerns, incorrect enrolment or perioperative change of surgical procedure (for example aborted surgery due to spread disease). After discussion in the study team, including statistician, patients converted from laparoscopic to open surgery generally stays in the study. Due to eCRF configuration, data can only be entered until postoperative day 2 for patients planned for laparoscopic surgery. In case of discontinuation, no further data will be registered in the eCRF except follow up of AEs and SAEs and 30-day mortality. Any saved blood samples

collected until then will be discarded, but data already entered into the eCRF will be included in the study analysis.

Discontinued subjects will not be replaced. The discontinued patients will be followed up and treated as long as needed by the treating physician, according to clinical routine, including treatment of SAEs and AEs.

4.5 Premature termination of the study

The Sponsor or the Investigators may decide to stop the complete study or part of the study at any time. If the study is prematurely terminated or suspended, the Investigators should promptly inform the previously included patients. Furthermore, the Investigators should promptly inform the Ethical Review Authority and provide a detailed written explanation. The Regulatory Authority should be informed according to national regulations.

No interim analysis is planned. The sponsor and the study group will review all Adverse Events, and Serious Adverse Events will be assessed for causality. This phase IV study is considered low risk, and no Data Safety Monitoring Board has been appointed.

5. Methodology

5.1 Treatment

Argipressin is the active substance in Empressin®. Patients will receive either continuous infusion of Empressin® 0.8 U/ml, or NaCl 9 mg/ml (placebo) at a constant rate of 0.056 ml/kg/h during surgery.

Table 2. Composition of the product

**Study Product**	**Concentration, dosage, administration**	**Manufacturer**
Empressin® 20 U/ml<br>Size of ampoule: 2 ml<br>(2 ml of Empressin® is mixed in 48 ml of NaCL 9 mg/ml, to a concentration of 0.8 U/ml).	Intravenous infusion of Empressin® 0.8 U/ml, 0.056 ml/kg/h (The solution is administered in a 50 ml syringe, by an infusion pump, in a central venous catheter). Infusion is started after induction of anesthesia, and stopped at the end of surgery.	AOP Orphan Pharmaceuticals AG<br>Färögatan 33, 31 TR<br>16454 Kista, Sweden<br>e-mail: office.se@aoporphan.com<br>phone: +46 0705786100
NaCl 9 mg/ml (“normal saline”) = placebo	Intravenous infusion of NaCl 9 mg/ml, 0.056 ml/kg/h (The solution is administered in a 50 ml syringe, by an infusion pump in a central venous catheter). Infusion is started after induction of anesthesia, and stopped at the end of surgery.	

5.1.1 Labelling

The study drug will be labelled with the Study Code (ARG-01), the EudraCT-number, "Study Drug", the subject number, route of administration, "for clinical study use only", name, address and telephone number to the investigator (**appendix D**). The number to the manufacturer and the batch number will be available in the drug preparation room, but not on the label of the syringe as to not jeopardize the blinding. The nurse preparing the syringe will sign it with date and signature.

5.1.2 Storage, handling and delivery

Commercially available Empressin® ampoules will be procured through the normal channels of distribution, i.e. hospital pharmacy at Sahlgrenska University hospital (or other hospital pharmacy, depending on study centre). The ampoules contain concentrated Empressin® which is very potent, and needs to be diluted before infusion. The Empressin® ampoules will be kept locked up at the drug preparation room in the anaesthesia department ("on the shelf"). After randomisation on the day of surgery, the assisting nurse will use one ampoule from the shelf for preparation of the study drug (if the patient is in the treatment group). The infusion of Empressin® needs to be prepared directly before use according to manufacturer's instructions, and hence it cannot be prepared by the hospital pharmacy in advance. The study nurse in cooperation with the OR staff will be responsible to keep the store refilled. The boxes with Empressin®-ampoules as well as NaCl-ampules used specifically for the study will be labelled with the study name, and the telephone number to the manufacturer, and to the principal investigator.

The labelling of the infusion syringe is performed when the content of an ampoule is mixed with saline in the syringe (**Appendix D**). The content of the infusion of Empressin® does not differ from the infusion content described in the IMPD (or FASS). The ampoules are labelled by the manufacturer, and this label is not changed or hidden during the study. The used ampoules are kept locked in the drug preparation room as to not interfere with the blinding, until assessed by the monitor.

Empressin® will be handled and prepared according to the instructions from the manufacturer in FASS. Should the date of expiry be passed (unmixed ampoules of Empressin® is durable for 3 years), it is the responsibility of the study nurse to alert the PI so that additional ampoules can be provided.

The placebo infusion consists of normal saline (NaCl 9 mg/ml) will be provided by the Anesthesia department at Sahlgrenska University Hospital. Any remaining infusion of study drug at the end of surgery will be disconnected from the patient and the syringe saved, after notation of the administered volume. The syringe will be discarded after inspection by the monitor.

It is the investigator's responsibility to establish a system for handling the study medicine to ensure that it is correctly received and recorded and only dispensed to patients in accordance with this protocol and the written manufacturer instruction in FASS. The empty ampoules of Empressin® and NaCl will be labelled with the subject number and saved in a dedicated box in the medical preparation room, and will only be discarded after inspection by the monitor.

5.1.3 Randomization, Blinding and Study Drug administration

Randomization in RedCap® and preparation of the study drug infusion syringe will be performed by an assisting nurse with access only to the randomization tool in Red Cap®. A list of assisting nurses with delegation to use the randomization tool in Red Cap® will be made. The syringe, labelled "Study drug", will be handed to the anesthesia nurse, who will be blinded to its content (both solutions are colorless and transparent), as well as the study staff and all healthcare professionals involved in the care of the patient. The assisting, unblinded, nurse will not be involved in the care of the patient.

The randomization tool in Red Cap® will generate information regarding weather active treatment or placebo will be given to the patient, as well as a randomization number. Allocation ratio will be 1:1. The randomization number will be noted on the anesthesia chart and in the medical record (Melior), and linked to an envelope with information about which treatment has been given. The envelopes are kept locked in, and are used only in case unblinding is needed due to any unexpected serious medical event jeopardizing the safety of the study participant. The attending physician is responsible for decisions regarding unblinding after discussion with the primary investigator or sponsor. Unblinding must be documented and reported to the investigator.

Treatment codes will not be broken for the planned analyses of data until all decisions on the evaluability of the data from each individual patient have been made and documented by the monitors at the end of the study (database lock).

5.1.4 Treatment compliance

As study treatment will be administered intravenously by hospital staff during surgery, and all samples will be obtained during hospitalization in conjunction with surgery, compliance is expected to be 100%.

5.1.5 Accountability

The study nurse will be responsible for collection of samples, to check that start and termination of the study drug is done at the correct timepoints, and ensure collection of perioperative data. Correct study drug administration is controlled by the monitor.

The study product provided for this study is for use only as directed in the protocol. All unused products will be accounted for and returned to the PI at the termination of the study. At the end of the study, it must be possible to reconcile delivery records with records of usage and returned batches.

5.2 Samples and Biobank

Haemoglobin, WBC, CRP, PLT, Albumin, plasma and urine-creatinine and hs-TNI, as well as AST, ALT and PK-INR will be analysed directly at the Department of Clinical Chemistry at Sahlgrenska University Hospital. When handled at the Department of Clinical Chemistry, the samples will be part of each centre's biobank. Analysis results are filed in the patient's hospital chart and then recorded in the eCRF under the "subject number".

Arterial lactate, glucose and S-Na will be obtained by point of care blood gas analysis, and the sample discarded after analysis. Analysis results are filed in the patient's hospital chart and then recorded in the eCRF under the "subject number". Urine [TIMP-2] x [IGFBP-7] will be analysed by point of care use of Nephrocheck®, and the result will be recorded directly in the eCRF. Samples for IFABP will be centrifugated immediately and kept at -80°C until the end of the study, when batch analysis will be performed at the Study section at the Department of Clinical Chemistry at Sahlgrenska University Hospital.

Plasma samples for interleukins and cytokines (IL-1β IL-6, IL-8, IL-10, MCP-1, SDF-1α, ICAM, C3a, C5b-9) will only be collected from patients included in the inflammatory subgroup. They will be handled by the research group/ study nurse, centrifugated immediately and stored at -80°C until batch analysis. These samples will be part of Sahlgrenska Biobank 809, Västra Götaland Region. From the serum drawn at each measurement point for the subgroup, one 500 microliter sample will be saved in case of future interest of supplemental analysis, although maximum for ten years. All samples will be discarded after thawing and analysis.

All stored samples will be handled according to current law (Swedish Code of Statutes 2002:297). The responsible investigator at each site keeps full tractability of collected biological samples from the subjects while in storage at the centre. Likewise, the sample receiver keeps full tractability of the samples while in storage and during use until used. The amount of blood drawn for study purposes, in addition to clinical routine samples, is 62.5 ml.

5.3 Concomitant medication

Concomitant medication during the study will be recorded in the eCRF (temporary omittance of medication in relation to surgery will be considered routine practice and not documented). Participants are allowed to continue with their regular medications during the study.

6. Study measurements

6.1 Measurements, time points and procedures

6.1.1 Preoperative visit

Screening

Written information about the study will be sent by e-mail or mail to each patient when summoned for the preoperative anesthesia visit, where screening for eligibility will take place. All patients, ASA class I-III, planned for elective hepatic surgery will be eligible for inclusion. Eligible patients will be given oral and written information about the study, and asked to provide a written consent, signed both by the patient and by appointed physicians at the preoperative center (a list of delegated physicians will be established). If the patient was not approached at the preoperative visit, screening and enrolment may also take place at admission to the surgical ward the day before surgery (with oral and written information provided by the research group, as well as the written consent form). In case information was not sent to the patient before the preoperative visit, written and oral information may be provided at the visit, by appointed physicians, and oral consent obtained by phone call. A signed written consent will then be obtained when the patient is admitted for surgery. After inclusion, the patient will be assigned a subject number. All patient data processed by the sponsor will be filed under this pseudonym, and the subject number will follow the patient throughout the study. ASA class IV patients (patients with significant life -threatening disease, such as recent cardiac infarction or major cardiac failure) will not be included in the study.

Before the preoperative visit, routine laboratory tests will be taken. This data is not specific for the study, and permission to include these results in the eCRF will be requested in the informed consent form. Demographic data, medical history and medications will also be recorded.

For the blood and urine- samples obtained during and after surgery, set times will be used for printed labels (preoperative samples; 8:00 am, end of surgery samples; 3:00 pm, end of surgery +3h samples; 6:00 pm, postoperative samples; 5:00 am). This routine makes it easier to distinguish the study samples in the chart. Samples at 10:00 am are not registered in LabBest, and no printed label is needed. The study nurse will note in the work sheet the actual time that samples were obtained, and a time span of ±1 hour is acceptable.

6.1.2 Day of surgery

Before anesthesia

Randomization in RedCap® and preparation of the study drug infusion syringe will be performed by an assisting (unblinded) nurse with limited access to the eCRF program (only the randomization module). The syringe, labelled "Study drug" (**Appendix D**), will be handed

to the anesthesia nurse, who thus will be blinded to its content (both solutions are colorless and transparent). The assisting nurse is not involved in the care of the patient.

Before induction of anesthesia, an arterial catheter will be inserted, and baseline blood samples obtained. Epidural catheter or intrathechal morphine for open surgery patients is placed before anesthesia at the discretion of the treating anesthesiologist.

After induction

After induction of anesthesia according to clinical routine, a central venous catheter and a urinary catheter will be inserted, and baseline urine samples will be obtained. A cardiac output monitor (Picco®) will be inserted at the discretion of the anesthesiologist (primarily for open surgery patients). Norepinephrine infusion (or other vasoactive drugs, such as nitroglycerine or dopamine) may be used as needed.

After collection of all baseline samples, the study drug or placebo is started in the central venous catheter, in a dedicated lumen, at a continuous rate of 0.056 ml/kg/h (0.045 U/kg/h, corresponding to 3.6 U/h for an 80-kg patient), and run until the end of surgery. The anesthesia nurse will alert the assisting nurse (who was responsible for randomization and preparation of the study drug) if an additional syringe will be needed in case off prolonged surgery. A maximum weight of 100 kg will be used for infusion rate calculation- obese patients will hence receive a slightly lower dose/kg to avoid excessively high doses.

Surgical procedure

Hepatic resection is carried out in different stages. In the first phase of surgery, the abdomen is dissected to access the liver (dissection phase), and then the resection of liver parenchyma ensues (resection phase).

The risk of blood loss is greatest during the resection phase, where liver parenchyma is divided, and occlusion of the hepatoduodenal ligament is applied intermittently by the surgeon to control blood loss. During the study, use of surgical occlusion techniques will be applied according to the preference of the surgeon, after discussion among the surgeons on a standardized routine. The resection area is also susceptible to the pressure in the central venous system, why anesthesiologic management aims to keep venous pressure low. Hemodynamic values, including CVP, will be assessed during surgery.

End of surgery

The study drug/placebo infusion will be given at a constant rate until the end of surgery, when it is tapered out by reduction with 50%, after 10 minutes further reduction with 50%, and after another 10 minutes discontinued. Norepinephrine may be used to counterbalance any fall in blood pressure.

Blood loss will be assessed by recording of the levels in suction devices used during surgery, counting of soaked gauze pieces, and deduction of ascites and irrigation fluid according to instructions given by the investigators. Utmost care will be taken to clear the abdomen of excess irrigation fluids during laparoscopic surgery, and amount of used irrigation fluid will be noted.

Samples will be obtained, as well as recordings of hemodynamic and perioperative data, such as unexpected surgical events. At the end of surgery, anesthesia is discontinued, the patient is extubated and transported to the postoperative care unit.

IFABP, Nephrocheck®, urine-creatinine and an arterial blood gas will be collected at 3 hours after conclusion of surgery, at the postoperative ward.

6.1.3 Postoperative procedures

Postoperative day 1

Routine blood samples are taken at approximately 5 am. The study nurse will obtain the tests that require immediate handling (centrifugation and freezing) in the morning, at approximately 10 am. Total volume of blood transfusion will be recorded, as well as urinary output and use of diuretics. If the arterial line is removed, a venous blood gas will be obtained.

Postoperative day 2

The patients treated with laparoscopic surgery are usually discharged from hospital at 2 days after surgery. Blood samples will be taken from all patients at this timepoint, both in the laparoscopic group as well as in the open surgery group.

Samples for analysis of interleukins and cytokines will be obtained in a subgroup of patients in each arm. These samples require immediate handling, and will obtained postoperative day 1 and 2.

Postoperative day 5

The open surgery group is usually discharged from hospital at 5 days after surgery. Blood samples will be taken at postoperative day 5 (or earlier if patient is discharged before postoperative day 5). Total transfusion requirements will be assessed at postoperative day 5 (or postoperative day 2, if the patient had laparoscopic surgery). Additional transfusions after this timepoint are not considered related to the study drug, and hence not registered in the eCRF.

6.1.4 Follow up

No extra visits or tests after postoperative day 2 (laparoscopic group) or day 5 (open surgery group) will be added due to the study. Patients will be followed up by the surgical team

according to clinical routine at 30 days after surgery. Usually, this follow-up is made by phone call, and postoperative complications will be registered in the SweLiv registry. Data will be obtained from the registry, and noted in the eCRF.

6.2 Recording of data

An electronic Case Registration Form (eCRF), Red Cap®, will be used for data collection. The investigators ensure the accuracy, completeness and timeliness of the data recorded, and will sign the completed eCRF after revision by the monitor. The complete Trial Master File, as well as source documents, will be archived for at least 15 years after the study is completed (or longer, if required by new Swedish regulations).

The treatment code will not be broken until the database has been cleaned, approved and locked by the sponsor, and all decisions on the evaluability of the data from each individual subject have been made and documented.

6.3 Study variables and laboratory analysis

All study data will be noted and recorded in the eCRF (Table 1). Some of the laboratory measurements are also safety parameters, and will be used both for assessment of the patient in clinical praxis, and in the study analysis.

6.3.1 Study variables

Background data

Sex, age, medical history, medication Clinical Frailty Scale, weight and height will be recorded, as well as indication for surgery.

Blood loss

Blood loss will be measured at the end of surgery by reading of amount of blood in suction devices, soaked gauze pieces and surgical dressings, according to instructions from the investigators. Utmost care will be taken to clear the abdomen of excess irrigation fluids during laparoscopic surgery, and amount of used irrigation fluid will be noted and subtracted. Volume of transfused blood will be recorded at the end of surgery, at postoperative day 1, and at postoperative day 2 and 5, respectively.

Descriptive data

As shown in Table 1, anaesthesiologic data (achievement of CVP goal, vasoactive medications, urinary output), hemodynamic data (heart rate, MAP, CVP and CI) and surgical data (performed surgical procedure, duration of surgery, time of surgical occlusion, radicality of resection, postoperative complications and length of stay) will be recorded.

6.3.2 Blood and urine

The total volume of blood needed for samples not included in routine management is 62.5 ml.

Plasma
CRP, Albumin, hs-TNI, Creatinine, AST, ALT and PT-INR will be analysed in plasma, as well as C3a and C5b-9. Cytokine samples will be centrifugated immediately and stored at -80°C for batch analysis.

Serum
IFABP and interleukins (IL-1β IL-6, IL-8, IL-10, MCP-1, SDF-1α, ICAM) will be analysed in serum. Samples will be centrifugated immediately and stored at -80°C for batch analysis.

Whole blood
Haemoglobin, WBC and PLT will be analysed in whole blood samples, as well as arterial lactate, glucose and Na (analysed by point of care blood gas analysis).

Urine
Samples of urine will be taken twice for analysis of [TIMP-2] x [IGFBP-7] and urine-creatinine.

6.3.3 Safety measurements and variables
The study trial will take place in hospital, and patients will be closely monitored prior to and after surgery according to clinical routine.

hs-TNI is analysed as part of the study, as well as plasma creatinine and arterial lactate; indicators of cardiac, renal and intestinal damage. Markers of inflammation and infection (WBC, CRP) are analysed both as part of the study and according to clinical routine. AST, ALT, PK-INR and PLT are analysed in clinical routine management and will be recorded in the eCRF. Results will be presented as descriptive/ explorative data.

Laboratory results indicating organ damage or need for surgical or medical treatment will be handled by the treating physician. This may include ECG and hs-TNI series if indicated, as well as consultation of other relevant specialists. Should complications to surgery arise, patients will be treated by the surgical team as long as required. No additional safety parameters due to the study will be assessed after postoperative day 2 (laparoscopic group) or postoperative day 5 (open surgery group).

All study related procedures will be performed according to current local and international safety recommendations to keep the risk for each participant as low as possible. In addition to the regular safety precautions, all participants will be observed for eventual AE as described above.

7. Safety

It is of the utmost importance that all staff involved in the study is familiar with the content of this section. The Principal Investigator is responsible for ensuring that procedures and expertise are available to handle medical emergencies during the study.

The term AE is used to include both serious and non-serious AEs. Detailed information on definitions and reporting rules are seen below in **Appendix C** (Definitions of Adverse Events).

All interventions of the study protocol will be performed according to local and international safety and procedural recommendations to assure the safety of the patient and to keep any risk as low as possible.

Safety surveillance and registration of Adverse Events (AE) and adverse reactions will be performed during the entire study. In case of SUSAR, the investigators will be alerted within 24 hours or the next working day, and decide if the study code is to be broken and potential causality investigated. Beyond the tests defined in the data collection protocol, no additional biochemical monitoring is considered necessary for this study. In case any of these tests indicate need for treatment, the treating physician will be responsible for assessment, treatment and follow up.

7.1 Expected adverse reactions

Pre-existing conditions prior to study start will not be registered as adverse reactions. The expected complication rate after hepatic resection varies between 4.1- 47.7 % (24). Common complications are hepatic failure, bile leakage, AKI, postoperative infection and more; such events will be noted in the eCRF, but considered part of the expected postoperative course, and not reason for unblinding. Also, expected adverse reactions to anaesthesia, such as hypotension, may be noted, but not considered an AE.

All vasoconstrictive drugs, including Argipressin, has the potential to affect perfusion of vital inner organs such as intestines and kidneys, as well as peripheral perfusion (skin and digits). The risk of hypoperfusion increases with higher doses, in combination with other vasopressors and in unstable, hypovolemic patients. The side effects Argipressin listed as “common” (>1/100) in FASS includes arrythmia, myocardial ischemia, peripheral vasoconstriction and digital ischemia, bowel hypoperfusion and stomach cramps. These conditions will be supervised with routine perioperative monitoring (ECG), per protocol assessment of digital capillary refill time, and by part of the laboratory results in this study.

Laboratory results outside of the normal range are considered AE:s if:
Hs-TNI: outside reference range

Plasma Creatinine: criteria for AKI fulfilled, i e baseline value x 1.5 or +27 micromoles/L
INR: > 1.4
AST and ALT: > baseline value x 20

7.2 Definitions of Adverse Events (AE)

An AE is the development of an undesirable medical condition or the deterioration of a pre-existing medical condition following or during exposure to a pharmaceutical product, whether or not considered causally related to the product. An undesirable medical condition can be symptoms (e.g., nausea, chest pain), signs (e.g., tachycardia, enlarged liver) or the abnormal results of an investigation (e.g., laboratory findings, electrocardiogram). In clinical studies, an AE can include an undesirable medical condition occurring at any time, from the date of signed consent, including run-in or washout periods, even if no study treatment has been administered, to the final follow-up visit (i.e postoperative day 2 (laparoscopic group) or postoperative day 5 (open surgery group)).
An AE will be followed up for as long as it is considered to be medically relevant.
It is important to distinguish between Serious Adverse Events (SAEs) and Severe AEs. Severity is a measure of intensity whereas seriousness is defined by the criteria listed below. An AE of severe intensity need not necessarily be considered serious.

7.2.1 Definition of severity rating

A definition for the grading of severity of AEs is provided below in Table 3.

Table 3 - Severity rating of an Adverse Event

0	None	No symptoms
1	Mild	Transient symptoms; awareness of sign or symptom, but easily tolerated.<br>No interference with the subject’s daily activities.
2	Moderate	Marked symptoms; discomfort sufficient to cause interference with normal activities.<br>Moderate interference with the subject’s daily activities.
3	Severe	Considerable interference with the subject’s daily activities; unacceptable, incapacitating.<br>Inability to perform normal activities.

7.2.2 Causal relationship

The following terms and definitions are used when assessing the causal relationship between each AE and the relevant study product (Table 4).

Table 4 - Casual relationship

1	Definite	There is no doubt that the incident is related.

2	Probable	Good reason and sufficient documentation to assume a causal relationship.
3	Possible	A causal relationship is conceivable and cannot be dismissed.
4	Unlikely	The event is most likely related to etiology other than the trial product.
5	Not related	The event is not related to the study product.
6	Unknown/Unclassifiable	A report suggesting an adverse event reaction, which cannot be judged because information is insufficient or contradictory and which cannot be supplemented.

7.3 Definitions of Serious Adverse Events

All SAEs must be collected, and an assessment of causality should be performed. An SAE is an event that results in one of the following:

- Results in death
- Is immediate life-threatening
- Requires in-patient hospitalisation or prolongation of existing hospitalization,
- Results in persistent or significant disability or incapacity,
- Is a congenital abnormality or birth defect,
- Is an important medical event that may jeopardize the subject or may require medical intervention to prevent one of the outcomes listed above

7.3.1 Recording and reporting of Adverse Events

All AEs will be collected throughout the study. It is the responsibility of the investigator to record and assess all AEs and SAEs until discharge from hospital (maximum postoperative day 5). The investigator have to alert the Principal Investigator to all SAEs within 24 hours after gaining knowledge about the AE/SAE (or on the next business day), and the Principal Investigator will then report the SAE to the sponsor. A supplementary report will follow with necessary information for a risk-benefit assessment.

7.3.2 Follow-up of unresolved Adverse Events

Any AEs that are unresolved at the end of the study are followed up by the treating physician (or study nurse, if delegated) for as long as medically indicated, but without further recording in the eCRF. A final outcome assessment will be made by the physicians in the research group. All AEs will be summarized and listed in the Clinical Study Report. SAE:s will also be described in all narratives/publications.

7.3.3 Suspected Unexpected Serious Adverse Reaction

A Suspected Unexpected Serious Adverse Reaction (SUSAR) is serious, according to the above criteria and is an untoward and unintended response to an investigational medical

product (IMP). The definition implies a reasonable possibility of a causal relationship between the event and the IMP. A serious adverse reaction, which is related to a non-IMP, is not a SUSAR and is not reported as such.

7.3.4 Reporting of Suspected Unexpected Serious Adverse Reaction

The sponsor is responsible for the immediate reporting of any serious adverse reaction that is suspected and unexpected to the European Database EudraVigilance and to the EC. Fatal and life-threatening events must be reported as soon as possible and no later than 7 calendar days after they have come to the sponsor's knowledge. A detailed written follow-up report with all relevant information must be submitted within an additional 8 days. For non-fatal and non-life-threatening SUSARs 15 days do apply. The causality of SAEs will be assessed. The Sponsor is responsible for SUSARs to be electronically reported and registered in the EudraVigilance database.

7.3.5 Reference safety information

The current reference safety information contained in the Product résumé (FASS) applies and constitutes the basis of the assessment if the adverse reaction is unexpected or not. The sponsor is responsible for the annual safety reporting including a summary of all SAEs and SUSARs that must be submitted to the SMPA and the EC as long as the study is ongoing.

The sponsor will report to the SMPA and EC, within 3 months after the study is completed, that the trial is ended as defined in the study protocol (Declaration of End of Trial Notification). After 12 months, a summary report will be register in the EudraCT database. The SMPA is requested to assist with the registrations in the EudraVigilance database and EudraCT database.

8. Study Management

8.1 Data Management

Study data will be collected using an electronic Case Report Form (eCRF; Red Cap®). All data handling activities will be described in the Data Management Plan. Clean File/database lock will be documented and dated. The reason for any excluded data or protocol deviations will be described in the study report.

8.2 Monitoring

The study will be monitored by an independent monitor before the study begins, during the study conduct, and after the study has been completed, so as to ensure that the study is carried out according to the protocol and that data is collected, documented, and reported according to ICH-GCP and applicable ethical and regulatory requirements. Monitoring is performed as per the study’s monitoring plan and is intended to ensure that the subject’s

rights, safety, and well-being are met as well as data in the eCRF are complete, correct, and consistent with the source data.

The monitor will have regular contacts with the study staff to verify informed consents of participating subjects, to confirm that facilities remain acceptable, that the investigational team is adhering to the protocol, that data are being accurately recorded in the eCRFs, and that all investigations are responsibly carried out according to the study protocol. The monitor will also ensure source data verification (comparison of the data in the eCRF with the hospital/practice and/or other records at the investigational site).

The Head of Department will allow the monitor transparency to the patient's medical records and other source documents and will provide subject confidentiality. The monitor must sign a confidentiality agreement.

All patient source data such as demographic data, medical history, clinical examinations and vital signs-readings and measurements analysis results from the hospital laboratories, will be stored in the patient files according to routines at the study sites.

8.3 Archiving

eCRF documentation and physical documentation including subject consents, study documents and other source data will be retained for at least 15 years after finalization of the study (or longer if required by Swedish regulations).
Information about the patients involved in the current study, the consent date, name of the study and name of the medical test product must be recorded, saved and stored according to national regulations in the hospital’s electronic medical records. The full pseudonymised dataset will be available from the corresponding author on reasonable request.

8.4 Quality assurance

The sponsor has the overall responsibility for the implementation of the clinical study. In the present study the sponsor will delegate all or part of their duties to the Principal Investigator at the Sahlgrenska University Hospital, and the investigators at each site. The Principal Investigator will ensure that there are written instructions for the study personnel, for the monitoring process, audits and inspections, that the personnel involved in the clinical trial has adequate competence and relevant training for their duties and that the subjects through insurance or otherwise have guaranteed adequate financial protection.
The responsible investigators at each site are responsible for the medical welfare of the study subjects, but may delegate responsibility for clinical treatment to the surgery team.

8.4.1 Audits and Inspections

Audits or inspections may be performed, including source data verification, in order to achieve a high quality of the study. The investigators will permit study-related monitoring,

audits, EC review, and regulatory inspection(s), providing direct access to source data/documents. The sponsor will allow transparency into the patient's medical records and other source documents and ensure subject confidentiality.

8.4.2 Significant changes

Significant changes in the protocol must be approved by the SMPA before they are implemented. The common Substantial Amendment Notification Form will be used. A substantial change is, for example, change of the main purpose, primary or secondary variables, method to measure the primary endpoint, change of investigational medicinal products or dosage.

8.5 Insurances

All participants of the study, as any other patient in the general Swedish health care, will be covered by the general patient insurance and pharmaceutical insurance.

9. Statistics

The complete SAP is attached as supplement 2. This single centre, double-blinded, randomized, placebo-controlled trial is performed at Sahlgrenska University Hospital. The aims are to investigate the effect of Argipressin on blood loss, transfusion requirements and inflammatory response during liver resection. Participants will be stratified according to planned surgical technique (open or laparoscopic surgery) and planned extent of resection (“large” or ”small”) before randomization to treatment with argipressin or placebo during surgery. Markers of kidney, cardiac and intestinal damage will be evaluated during and after surgery. More extensive testing of inflammatory markers will be performed in a limited number of patients (subgroup) in each arm.

Analysing models

All analyses will be performed using SAS software version 9.4 or later (SAS Institute Inc., Cary, NC, USA).
Descriptively, continuous variables will be described by mean, SD, median, interquartile range, and range as appropriate, and categorical variables by number and percentage.
For test between two groups Fisher's exact test will be used for dichotomous variables, Mantel-Haenszel Chi-square trend test for ordered categorical variables, Chi-square test for non-ordered categorical variables, and two-sample t-test or Mann-Whitney U-test for continuous variables as appropriate based on their distribution.
Analysis of normally distributed outcome variables will be performed using general linear model adjusted for randomization strata (laparoscopic, open small and open large surgeries), and those that are lognormally distributed generalized linear model will be used with lognormal distribution with the same adjustment. In case of not fulfilling model assumptions non-parametric unadjusted Mann-Whitney U-test will be used for continuous

outcome variables. Dichotomous variables will be analysed using binary logistic regression adjusted for randomization strata as above. Number of complications per follow-up time will be analysed using Poisson regression including adjustment for randomization strata. Time-to-event data, such as length of stay in hospital, will be graphically described using Kaplan-Meier technique and analysed using Cox regression model, with the same adjustment. Assumption of proportional hazard will be checked by studying interactions with time in study. From these analyses, difference in least square means, relative risk (RR), odds-ratio (OR), and hazard ratio (HR) with 95% CI between the treatment groups and associated p-values will be the main measures presented.

The secondary variables that will be included in the confirmatory fixed sequential testing per the order below following the confirmation of the primary analysis, with 0.05 significance level, will be:

- The volume of blood transfusions during surgery and hospitalization
- The proportion of patients receiving blood transfusion

All other secondary endpoints will be evaluated in exploratory manner, and hence not adjusted for multiplicity. All exploratory tests will be cautiously considered, considering the magnitude and clinical relevance of the results.
Tests for interleukin and cytokine levels (IL-1b IL-6, IL-8, IL-10, MCP-1, SDF-1a, ICAM, C3a, C5b-9) will only be analysed in a subgroup of 44 patients in each parallel group (total n=88). The results will be presented as explorative data.

Sample size
Calculation of sample size was performed based on data from open and laparoscopic liver resections performed at Sahlgrenska University Hospital during the period Jan 2019-Oct 2020. When applying log-transformation on the blood volume variable normal distribution was obtained. The sample size calculation was performed using two-sided t-test on the log-transformed variable that will correspond to the selected method for the primary analysis, i.e. generalized linear model with lognormal distribution. To be able to find at least 35% reduction in the mean blood loss in the treatment arm compared to the placebo arm (962 ml vs 625 ml, respectively), i.e. a difference of at least 0.43 in the log-scale assuming SD of 1.17, with a level of significance of 5%, and a power of 80%, 118 patients are needed to be included in each arm. Assuming a dropout rate of 5%, 248 patients in total will need to be included, 124 patients in each arm. A limited number of patients will be discovered fulfilling the exclusion criteria after the randomization and first after they have started the surgery. Since the investigational product is given from the start of the surgery these patients will be part of the Safety population and their safety outcome regarding 30-day survival will be collected. Their surgery will be immediately stopped, and they will be excluded from the study and the ITT population from all efficacy analyses. To account for these dropouts 248 ITT evaluable patients will be included, but not more than 272 randomized patients in total.

For the subgroup analysis 44 patients in the argipressin and placebo group respectively, will be included (total n= 88). The number of patients selected for extended inflammatory testing is based on a pilot study (unpublished data) performed at Sahlgrenska University Hospital.

Randomization

Participants will be stratified according to planned surgical technique (open or laparoscopic surgery) and planned extent of resection ("large" or "small") before randomization to treatment with argipressin or placebo during surgery. "Small resection" = wedge resections with maximum 4 resections, or segmental resections, maximum 2 segments. "Large resection" = More than 2 segments if anatomical resections, or resections including more than 4 segments (wedge or segmental resections, including right or left lobe resections and extended resections).

Safety

All adverse events (AE) from the first visit onwards will be collected in the study. Treatment emergent AEs are those that have started on or after the first dose of study drug provided in the study, and will be summarized. AEs occurring prior to the provided study intervention will also be tabulated. The AEs will be collected continuously during the study and will be coded using Medical dictionary for regulatory activities (MedDRA) to standardized system organ class (SOC) and preferred term (PT) before summarized.

10. Report and publication

The main results of the study analyses and physiological measurements will be evaluated and reported after all parts of the study data having been collected, compiled and evaluated. The investigators will only have access to blinded data until after termination of the study and data base lock. There will be a clinical study report, based on the main results including safety data, regardless of the results and without restrictions.

The results of the trial may be published by the investigators in an international scientific journal and presented at international meetings. Authorship will be granted depending on contribution to the study, according to the ICMJE guidelines.

11. Study timetable

First subject in: February 2022
Last subject finalized: December 2024

11.1 Definition of "End of study"

The end of the entire study is defined as "the last performed investigation in the last subject". In the present study this will be when data from the 30 day follow up is collected for the last patient included.

12. Declaration of interests and funding

This work was supported by the Department of Anaesthesia and Intensive Care, Sahlgrenska University Hospital, and the following grants: Gothenburg University, and the Sahlgrenska University Hospital (ALF-LUA), Gothenburg Medical Society (GLS-974156, GLS-961705), Sahlgrenska University Funds (SU-961353), Anna-Lisa Bror Björnsson Foundation, Tore Nilson's Foundation, and The Foundation for Transplantation and Cancer Research.

This is an investigator-initiated trial, and the authors have no conflicts of interest. The funders have no role in study design, analysis, preparation of manuscript or publication procedure.

13. References

1. Katz SC, Shia J, Liau KH, Gonen M, Ruo L, Jarnagin WR, et al. Operative blood loss independently predicts recurrence and survival after resection of hepatocellular carcinoma. Ann Surg. 2009;249(4):617-23.
2. Kooby DA, Stockman J, Ben-Porat L, Gonen M, Jarnagin WR, Dematteo RP, et al. Influence of transfusions on perioperative and long-term outcome in patients following hepatic resection for colorectal metastases. Ann Surg. 2003;237(6):860-9; discussion 9-70.
3. Margonis GA, Kim Y, Samaha M, Buettner S, Sasaki K, Gani F, et al. Blood loss and outcomes after resection of colorectal liver metastases. J Surg Res. 2016;202(2):473-80.
4. Wang CC, Iyer SG, Low JK, Lin CY, Wang SH, Lu SN, et al. Perioperative factors affecting long-term outcomes of 473 consecutive patients undergoing hepatectomy for hepatocellular carcinoma. Ann Surg Oncol. 2009;16(7):1832-42.
5. Wagener G, Gubitosa G, Renz J, Kinkhabwala M, Brentjens T, Guarrera JV, et al. Vasopressin decreases portal vein pressure and flow in the native liver during liver transplantation. Liver Transpl. 2008;14(11):1664-70.
6. Wisen E, Svennerholm K, Sand Bown L, Houltz E, Rizell M, Lundin S, et al. Vasopressin and nitroglycerin decrease portal and hepatic venous pressure and hepato-splanchnic blood flow. Acta Anaesthesiol Scand. 2018.
7. Pietsch UC, Herrmann ML, Uhlmann D, Busch T, Hokema F, Kaisers UX, et al. Blood lactate and pyruvate levels in the perioperative period of liver resection with Pringle maneuver. Clin Hemorheol Microcirc. 2010;44(4):269-81.
8. Lautt WW. Regulatory processes interacting to maintain hepatic blood flow constancy: Vascular compliance, hepatic arterial buffer response, hepatorenal reflex, liver regeneration, escape from vasoconstriction. Hepatol Res. 2007;37(11):891-903.
9. Abbas MS, Mohamed KS, Ibraheim OA, Taha AM, Ibraheem TM, Fadel BA, et al. Effects of terlipressin infusion on blood loss and transfusion needs during liver resection: A randomised trial. Acta Anaesthesiol Scand. 2019;63(1):34-9.
10. Sims CA, Holena D, Kim P, Pascual J, Smith B, Martin N, et al. Effect of Low-Dose Supplementation of Arginine Vasopressin on Need for Blood Product Transfusions in Patients With Trauma and Hemorrhagic Shock: A Randomized Clinical Trial. JAMA Surg. 2019;154(11):994-1003.
11. Schwarz C, Fitschek F, Bar-Or D, Klaus DA, Tudor B, Fleischmann E, et al. Inflammatory response and oxidative stress during liver resection. PLoS One. 2017;12(10):e0185685.
12. Evans C, Dalgleish AG, Kumar D. Review article: immune suppression and colorectal cancer. Aliment Pharmacol Ther. 2006;24(8):1163-77.
13. Russell JA, Walley KR. Vasopressin and its immune effects in septic shock. J Innate Immun. 2010;2(5):446-60.
14. Mukhtar A, Mahmoud I, Obayah G, Hasanin A, Aboul-Fetouh F, Dabous H, et al. Intraoperative terlipressin therapy reduces the incidence of postoperative acute kidney injury after living donor liver transplantation. J Cardiothorac Vasc Anesth. 2015;29(3):678-83.
15. Bragadottir G, Redfors B, Nygren A, Sellgren J, Ricksten SE. Low-dose vasopressin increases glomerular filtration rate, but impairs renal oxygenation in post-cardiac surgery patients. Acta Anaesthesiol Scand. 2009;53(8):1052-9.

16. Fayed N, Refaat EK, Yassein TE, Alwaraqy M. Effect of perioperative terlipressin infusion on systemic, hepatic, and renal hemodynamics during living donor liver transplantation. J Crit Care. 2013;28(5):775-82.
17. Russell JA, Walley KR, Singer J, Gordon AC, Hebert PC, Cooper DJ, et al. Vasopressin versus norepinephrine infusion in patients with septic shock. N Engl J Med. 2008;358(9):877-87.
18. Gordon AC, Mason AJ, Thirunavukkarasu N, Perkins GD, Cecconi M, Cepkova M, et al. Effect of Early Vasopressin vs Norepinephrine on Kidney Failure in Patients With Septic Shock: The VANISH Randomized Clinical Trial. JAMA. 2016;316(5):509-18.
19. Writing Committee for the VSI, Devereaux PJ, Biccard BM, Sigamani A, Xavier D, Chan MTV, et al. Association of Postoperative High-Sensitivity Troponin Levels With Myocardial Injury and 30-Day Mortality Among Patients Undergoing Noncardiac Surgery. JAMA. 2017;317(16):1642-51.
20. Rhodes A, Evans LE, Alhazzani W, Levy MM, Antonelli M, Ferrer R, et al. Surviving Sepsis Campaign: International Guidelines for Management of Sepsis and Septic Shock: 2016. Intensive Care Med. 2017;43(3):304-77.
21. Mukhtar A, Salah M, Aboulfetouh F, Obayah G, Samy M, Hassanien A, et al. The use of terlipressin during living donor liver transplantation: Effects on systemic and splanchnic hemodynamics and renal function. Crit Care Med. 2011;39(6):1329-34.
22. Bown LS, Ricksten SE, Houltz E, Einarsson H, Sondergaard S, Rizell M, et al. Vasopressin-induced changes in splanchnic blood flow and hepatic and portal venous pressures in liver resection. Acta Anaesthesiol Scand. 2016;60(5):607-15.
23. Derikx JP, Schellekens DH, Acosta S. Serological markers for human intestinal ischemia: A systematic review. Best Pract Res Clin Gastroenterol. 2017;31(1):69-74.
24. Ishii M, Mizuguchi T, Harada K, Ota S, Meguro M, Ueki T, et al. Comprehensive review of post-liver resection surgical complications and a new universal classification and grading system. World J Hepatol. 2014;6(10):745-51.

Appendix A - Signatures

SPONSOR'S AND INVESTIGATOR'S SIGNATURES

I hereby certify that the study will be conducted in accordance with the Study Protocol and applicable regulations, SMPA regulation 2011:19, ICH-GCP and latest version of the Declaration of Helsinki (2013):

Kristina Svennerholm<br>Department of Anaesthesia and Intensive Care<br>Sahlgrenska University Hospital<br>413 45 Gothenburg, Sweden	Date (20211027)
Peter Dahm<br>Head of the Department of Anaesthesia and Intensive Care<br>Sahlgrenska University Hospital<br>413 45 Gothenburg, Sweden	Date (20211027)

Appendix B - Ethics and regulatory review

The final study protocol, including the final version of the Written Informed Consent Form and other information given to subjects (as advertisements) must be approved or given a favourable opinion by an EC before enrolment of any subject into the study.
Before enrolment of any subject into the study, the final study protocol, including the final version of the Informed Consent Form, is approved by the national regulatory authority or a notification to the national regulatory authority is done, according to local regulations.
It is the responsibility of the Principal Investigator to apply to the EC in writing. The application document should:

- contain the name and address of the EC,
- clearly identify, by title and date, the protocol and other documents submitted for review, and
- be dated.

In addition, the Principal Investigator should request the EC to provide:

- their approval/opinion in a dated document identifying the Principal Investigator’s application,
- EC composition for the meeting when the approval was given, and
- a statement confirming that the EC is organised and operates according to GCP and applicable laws and regulations.

The Principal Investigator is responsible for informing the EC of any modifications and amendments to the protocol as per local requirements.
Progress reports and notifications of serious and unexpected adverse drug reactions will be provided to the EC according to local regulations and guidelines.
The Principle Investigator should file all correspondence with the EC in the appropriate form.
The Principle Investigator is also responsible for obtaining approvals from scientific bodies (eg, to use radiolabelled substances) if necessary for the study.

SUBJECT INFORMATION AND WRITTEN INFORMED CONSENT FORM

The Principle Investigator will ensure that the subject is given full and adequate oral and written information about the nature, purpose, possible risk and benefit of the study. Subjects must also be notified that they are free to discontinue from the study at any time. The subject should be given the opportunity to ask questions and allowed time to consider the information provided.
The subject’s signed and dated informed consent must be obtained before conducting any procedure specifically for the study.
The Principal Investigator must store the original, signed Informed Consent Form in the Investigator’s Study File. A copy of the signed Informed Consent Form must be given to the subject.
If a protocol amendment requires a change to the Informed Consent Form, the EC must approve modifications that lead to a revised Informed Consent Form before the revised form is used.

The content of the informed consent form complies with relevant integrity and data protection legislation. In the subject information and the informed consent form, the subject will be given complete information about how collection, use and publication of their study data will take place. The subject information and the informed consent form will explain how study data are stored to maintain confidentiality in accordance with national data legislation (please describe how data is stored and which data security measures are taken). All information processed by the sponsor will be pseudonymized and identified with <<Subject number>>.

The informed consent form will also explain that for verification of the data, authorized representatives of the sponsor, as well as relevant authority, may require access to parts of medical records or study records that are relevant to the study, including the subject’s medical history.

Subject data protection

The Informed Consent Form will incorporate (or, in some cases, be accompanied by a separate document incorporating) wording that complies with relevant data protection and privacy legislation.
Extra precautions are taken to preserve confidentiality and prevent genetic or other study data being linked to the identity of the subject. In exceptional circumstances, however, certain individuals might see both the genetic and/or other study data as well as the personal identifiers of a subject. For example, in the case of a medical emergency or an investigator might know a subject’s identity and also have access to his or her data. Also, Regulatory

authorities may require access to the relevant files, though the subject's medical information and the genetic files would remain physically separate.

Audits and inspections

Authorized representatives of the Sponsor, a regulatory authority, or an EC may perform audits or inspections at the centre, including source data verification. The purpose of an audit or inspection is to systematically and independently examine all study-related activities and documents, to determine whether these activities were conducted, and data were recorded, analysed, and accurately reported according to the protocol, GCP, guidelines of the ICH, and any applicable regulatory requirements.

Appendix C - Definitions of Adverse Events

Definitions:
- Adverse Events
- Serious Adverse Events
- Definitions of severity rating
- Causal relationship
- Action taken
- Reporting in CRF
- Adverse Events based on signs and symptoms
- Follow up and outcome assessment
- Suspected Unexpected Serious Adverse Reaction (SUSAR)

Further guidance on Serious Adverse Events:
- Life threatening
- Hospitalisation
- Important medical event or medical intervention
- A guide to interpreting the causality QUESTION
- Other significant Adverse Events
- Overdose

DEFINITIONS OF ADVERSE EVENTS AND PROCEDURES IN CASE OF PREGNANCY

It is of the utmost importance that all staff involved in the study is familiar with the content of this section. The Principal Investigator is responsible for ensuring this.

Adverse Events

An AE is the development of an undesirable medical condition or the deterioration of a pre-existing medical condition following or during exposure to a pharmaceutical product, whether or not considered causally related to the product. An undesirable medical condition can be symptoms (e.g., nausea, chest pain), signs (e.g., tachycardia, enlarged liver) or the abnormal results of an investigation (e.g., laboratory findings, electrocardiogram). In clinical studies, an AE can include an undesirable medical condition occurring at any time, from the date of signed consent, including run-in or washout periods, even if no study treatment has been administered, to the final follow-up visit.
An AE will be followed up for as long as it is considered to be medically relevant.
It is important to distinguish between SAEs and severe AEs. Severity is a measure of intensity whereas seriousness is defined by the criteria listed below. An AE of severe intensity need not necessarily be considered serious.

Serious Adverse Events

It is important to distinguish between SAEs and severe AEs. Severity is a measure of intensity whereas seriousness is defined by the criteria listed below. An AE of severe intensity need not necessarily be considered serious. For example, nausea which persists for several hours may be considered severe nausea, but not an SAE. On the other hand, a stroke, which results in only a limited degree of disability may be considered a mild stroke but would be a SAE.
<u>A medical emergency usually constitutes an SAE and is to be reported as such.</u>
A SAE is an AE occurring during any study phase (i.e. run-in, pre-entry, screening, treatment, wash-out, follow-up), and at any dose of the investigational product, comparator or placebo, that fulfils one or more of the following criteria:

- results in death,
- is immediately life-threatening,
- requires in-patient hospitalisation or prolongation of existing hospitalisation,
- results in persistent or significant disability or incapacity,
- is a congenital abnormality or birth defect,
- is an important medical event that may jeopardise the subject or may require medical intervention to prevent one of the outcomes listed above.

Definitions of severity rating

0	None	No symptoms
1	Mild	**Transient symptoms; awareness of sign or symptom, but easily tolerated.**<br>**No interference with the subject's daily activities.**
2	Moderate	Marked symptoms; discomfort sufficient to cause interference with normal activities.<br>Moderate interference with the subject's daily activities.
3	Severe	**Considerable interference with the subject's daily activities; unacceptable, incapacitating.**<br>**Inability to perform normal activities.**

Causal relationship

The causality of (S)AEs (ie, their relationship to study treatment and/or the investigational procedure) will be assessed by the investigator(s), who in completing the relevant case report form must answer "yes" or "no" to the question "Do you consider that there is a reasonable possibility that the event may have been caused by the drug/the investigational procedure?". The following terms and definitions are used when assessing the causal relationship between each AE and the relevant trial product(s):

1	Definite	There is no doubt that the incident is related.
2	Probable	**Good reason and sufficient documentation to assume a causal relationship.**
3	Possible	A causal relationship is conceivable and cannot be dismissed.
4	Unlikely	The event is most likely related to aetiology other than the trial product.
5	Not related	The event is not related to the study product.
6	Unknown/Unclassifiable	A report suggesting an adverse event reaction, which cannot be judged because information is<br>insufficient or contradictory and which cannot be supplemented.

Causal relationship in cases where the disease under study has deteriorated due to lack of effect will be classified as no reasonable possibility.

For SAEs causal relationship will also be assessed for additional study drug and/or other medication and/or study procedure. Note that for SAEs that could be associated with any study procedure the causal relationship is implied as "yes".

Action taken:

- None
- Dose of study drug changed
- Study drug temporarily stopped
- Study drug stopped

Reporting in the Case Report Form

The following variables will be recorded in the CRF for each AE: description of the AE, the date and time when the AE started and stopped, maximum intensity, whether the AE is serious or not, causality rating (yes or no; if yes specify), action taken with regard to investigational product, AE caused subject to discontinue study and outcome.

Adverse Events based on signs and symptoms

All AEs spontaneously reported by the subject or reported in response to the open question from the study personnel: *"Have you had any health problems since the previous visit?"*, or revealed by observation will be collected and recorded in the CRF. When collecting AEs the recording of diagnoses is preferred (when possible) to recording a list of signs and symptoms. However, if a diagnosis is known and there are other signs or symptoms that are not generally part of the diagnosis, the diagnosis and each sign or symptom will be recorded separately.

Follow-up - Outcome assessment

Any AEs that are unresolved at the patient's last AE assessment in the study are followed up by the investigators for as long as medically indicated, but without further recording in the CRF. The following terms and definitions are used in assessing the final outcome of an AE:

1. Recovered - The subject has fully recovered, or by medical or surgical treatment the condition has returned to the level observed at the first trial-related activity after the subject signed the informed consent.

2. Recovering - This term is only applicable if the subject has completed the trial or has died from another AE. The condition is improving and the subject is expected to recover from the event.

3. Recovered with sequelae - The subject has recovered from the condition, but with lasting effect due to a disease, injury, treatment or procedure. If a sequele meets an SAE criterion, the AE must be reported as an SAE.

4. Not recovered - The condition of the subject has not improved and the symptoms are unchanged, or the outcome is not known at the time of reporting.

5. Fatal - This term is only applicable if the subject died from a condition related to the reported AE. Outcomes of other reported AEs in a subject before he/she died should be assessed as “recovered”, “recovering”, “recovered with sequelae” or “not recovered”. An AE with fatal outcome must be reported as an SAE.

6. Unknown - This term is only applicable if the subject is lost to follow-up.

Suspected Unexpected Serious Adverse Reaction

For studies in countries implementing the EU Clinical Trials Directive, informing ECs and Regulatory Authorities will be performed by the sponsor.
In the case of research with a medical product there may be a causal link to the administered product. If it concerns an unexpected serious reaction, then this is a SUSAR.

Definition of a SUSAR:

1. The event must be **serious**, that is to say irrespective of the dose the event is:
   - fatal, and/or
   - life-threatening for the research subject, and/or
   - makes hospital admission or an extension of the admission necessary, and/or
   - causes persistent or significant invalidity or work disability, and/or
   - manifests itself in a congenital abnormality or malformation.
2. There must be a certain degree of probability that the event is harmful, and an undesirable, reaction to the medicinal product being investigated, regardless of the administered dosage. In other words, there is an adverse reaction.
3. The adverse reaction must be unexpected. That is to say, the nature and severity of the adverse reaction are not in agreement with the product information as recorded in:
   - for an authorised medicinal product: the SPC text (Summary of Product Characteristics: in the Netherlands this is the IB1 text). In the case of an international multicentre research with an authorised product, the sponsor may choose an SPC. If there is, in comparison to the authorisation, a different administration form, indication, patient group or dose, then the summary of the product information is supplemented with the relevant supplemental information for the concerned study.
   - for an unauthorised medicinal product: the Investigator’s Brochure/Product résumé.

Reporting of SUSAR

SUSARs within the study that are life threatening or have had fatal consequences must be reported, at the latest, within 7 days after the sponsor has become aware of them. All relevant information on the aftermath of this must be reported within a time period of a further 8 days. Other reports of SUSARs must be made within 15 days after the sponsor has become aware of them.

The Clinical Study Serious Adverse Event Report Form will be used together with other relevant supporting documentation (e.g. ECG, laboratory results, autopsy report) and relevant CRF modules. All SUSARs have to be electronically registered in the EudraVigilance database.

FURTHER GUIDELINES ON THE DEFINITION OF A SERIOUS ADVERSE EVENT

Life threatening

'Life threatening' means that the subject was at immediate risk of death from the AE as it occurred or it is suspected that use or continued use of the product would result in the subject's death. 'Life threatening' does not mean that if an AE had occurred in a more severe form it might have caused death (eg, hepatitis that resolved without hepatic failure).

Hospitalisation

Out-patient treatment in an emergency room is not in itself a SAE, although the reasons for it may be (eg, bronchospasm, laryngeal oedema). Hospital admissions and/or surgical operations planned before or during a study are not considered AEs if the illness or disease existed before the subject was enrolled in the study, provided that it did not deteriorate in an unexpected way during the study.

Important medical event or medical intervention

Medical and scientific judgement should be exercised in deciding whether a case is serious in situations where important medical events may not be immediately life threatening or result in death, hospitalisation, disability or incapacity but may jeopardize the subject or may require medical intervention to prevent one or more outcomes listed in the definition of serious. These should usually be considered as serious.

Examples of such events are:

- *Angioedema not severe enough to require intubation but requiring iv hydrocortisone treatment.*
- *Hepatotoxicity caused by paracetamol (acetaminophen) overdose requiring treatment with N-acetylcysteine.*
- *Intensive treatment in an emergency room or at home for allergic bronchospasm.*
- *Blood dyscrasias (eg, neutropenia or anaemia requiring blood transfusion, etc) or convulsions that do not result in hospitalisation.*
- *Development of drug dependency or drug abuse.*

A guide to interpreting the causality QUESTION

The following factors should be considered when deciding if there is a "reasonable possibility" that an AE may have been caused by the drug.

- Time Course. Exposure to suspect drug. Has the subject actually received the suspect drug? Did the AE occur in a reasonable temporal relationship to the administration of the suspect drug?
- Consistency with known drug profile. Was the AE consistent with the previous knowledge of the suspect drug (pharmacology and toxicology) or drugs of the same pharmacological class? OR could the AE be anticipated from its pharmacological properties?
- Dechallenge experience. Did the AE resolve or improve on stopping or reducing the dose of the suspect drug?
- No alternative cause. The AE cannot be reasonably explained by aetiology such as the underlying disease, other drugs, other host or environmental factors.
- Rechallenge experience. Did the AE reoccur if the suspected drug was reintroduced after having been stopped? A re-challenge would not normally be recommended or supported.
- Laboratory tests. A specific laboratory investigation (if performed) has confirmed the relationship?

A “reasonable possibility” could be considered to exist for an AE where one or more of these factors exist. In contrast, there would not be a “reasonable possibility” of causality if none of the above criteria apply or where there is evidence of exposure and a reasonable time course but any dechallenge (if performed) is negative or ambiguous or there is another more likely cause of the AE.
In difficult cases, other factors could be considered such as:

- Is this a recognised feature of overdose of the drug?
- Is there a known mechanism?

Ambiguous cases should be considered as being a “reasonable possibility” of a causal relationship unless further evidence becomes available to refute this. Causal relationship in cases where the disease under study has deteriorated due to lack of effect should be classified as no reasonable possibility.

Overdose

An overdose with associated AEs is recorded as the AE diagnosis/symptoms on the relevant.

Appendix D - Marking of syringes

ARG-01	**STUDY DRUG**<br>Empressin® 0.8U/ml /placebo	
EudraCT No. 2021001806-32<br>Contacts:	Subject number:	
Kristina Svennerholm, AnOpIva Sahlgrenska, tel 0708117993<br>Ellinor Wisén, AnOpIva Sahlgrenska tel: 0702457661	For clinical study use only	For intravenous use
