# Supplementary material for: Argipressin for prevention of blood loss during liver resection: a study protocol for a randomised, placebo-controlled, double-blinded trial (ARG-01)
Source: BMJ Open. 2023 Aug 24;13(8):e073270. doi: 10.1136/bmjopen-2023-073270 (PMC10450082; doi:10.1136/bmjopen-2023-073270)
Supplement: Supplementary data [file bmjopen-2023-073270supp002.pdf]

Primary registry and trial identifying number	ClinicalTrials.gov; NTC05293041
Date of registration in primary registry	Nov 15, 2021
Secondary identifying numbers	EudraCT 2021-001806-32
Source(s) of monetary or material support	Department of Anaesthesia and Intensive Care, Sahlgrenska University Hospital.<br>Gothenburg University, and the Sahlgrenska University Hospital (ALF-LUA),<br>Gothenburg Medical Society (GLS-974156, GLS-961705),<br>Sahlgrenska University Funds (SU-961353),<br>Anna-Lisa Bror Björnsson Foundation,<br>Tore Nilson's Foundation,<br>The Foundation for Transplantation and Cancer Research
Primary sponsor	Region Vastra Gotaland,
Secondary sponsor(s)	Kristina Tempelman Svennerholm
Contact for public queries	Ellinor Wisén, MD, +46 702457661<br>ellinor.wisen@vgregion.se
Contact for scientific queries	Kristina Tempelman Svennerholm<br>+46 708117993<br>kristina.svennerholm@vgregion.se
Public title	Argipressin for prevention of blood loss during liver resection
Scientific title	Influence of Argipressin on blood loss during hepatic resection; a double-blinded, randomized placebo-controlled trial.
Countries of recruitment	Sweden
Health condition(s) or problem(s) studied	Hepatic resection, colorectal cancer, hepatocellular cancer, gallbladder cancer, Klatskin tumour, liver metastasis, inflammation, transfusion
Intervention(s)	Active comparator: Empressin® 0.8 U/ml, 0.045 U/kg/h (0.056 ml/kg/h)<br>Placebo comparator: Normal Saline (NaCl 9 mg/ml)
Key inclusion and exclusion criteria	*Ages eligible for study*: ≥18 years<br>*Sexes eligible for study*: both<br>*Accepts healthy volunteers*: No<br>*Inclusion criteria*: patient ≥18 years, ASA class I-III, scheduled for open or

	laparoscopic hepatic resection, signed informed consent<br>*Exclusion criteria*:<br>-Participant does not understand the given information, and/ or cannot give written informed consent.<br>-Simultaneous operation of tumor with other localization, or surgery for superficial single hepatic tumor less than 2 cm, expected to be of short duration and with minimal blood loss.<br>-Terminal kidney failure (estimated preoperative GFR< 15 ml/min).<br>Pregnancy or lactation.<br>-Known allergy to Empressin®.<br>-Patient included in other interventional study, interacting with the endpoints in the present study, or previous randomization in this study.<br>-Hyponatremia (S-Na < 130 mmol/L)<br>-Patient considered ineligible for other surgical or medical reason.<br>-Present infection.<br>Patients with systemic inflammatory disease, inflammatory bowel disease or preoperative corticosteroid treatment will not be eligible for the subgroups where cytokines and interleukins are investigated.
Study type	Interventional<br>Allocation: randomized<br>Intervention model: 2 parallell arms<br>Masking: Double blinded (subject, caregiver, investigator, outcomes assessor)<br>Primary purpose: morbidity reduction<br>Phase: IV
Date of first enrolment	March, 2022
Target sample size	248 patients
Recruitment status	recruiting
Primary outcome(s)<br>Key secondary outcomes	*Primary outcome*: Blood loss (ml) at the end of surgery<br><br>*Secondary outcomes*:<br>Transfusion needs during surgery and postoperative day 1, 2 (laparoscopic surgery) or 5 (open surgery).

	Laboratory inflammatory response after hepatic resection.<br><br>Change in organ damage markers
