# Supplementary material for: Argipressin for prevention of blood loss during liver resection: a study protocol for a randomised, placebo-controlled, double-blinded trial (ARG-01)
Source: BMJ Open. 2023 Aug 24;13(8):e073270. doi: 10.1136/bmjopen-2023-073270 (PMC10450082; doi:10.1136/bmjopen-2023-073270)
Supplement: Supplementary data [file bmjopen-2023-073270supp003.pdf]

Table 1. Study measurement points and time frame

	Enrolment	**Allocation**	**Intervention**			**Post operative care →**				***Close -out***
**TIMEPOINT**	***Pre op visit***	**Day of surgery -** *Before anesthesia*	**Day of Surgery-** *After induction*	**Day of Surgery-** *End of surgery*	**Day of Surgery-** End of Surgery +3h	***Post op day 1*** *5 am*	***Post op day 1*** *10 am*	***Post op day 2***	***Post op day 5****	***30 day follow up***
**ENROLMENT:**										
Eligibility screen	X									
Informed consent	X									
Demographic data	X									
Allocation		X								
**INTERVENTION:**										
Study drug			I--------------	-----------I						
assessment of AEs		X	X	X	X	X	X	X	X	
**ASSESSMENTS:**										
Blood loss				X						
Blood transfusion (ml)				X			X	X	X	
Norepinephrine**				X						
Use of other inotropic drugs				X						
CVP-goal achieved				X						
Total diuresis							X			
Furosemide total dose							X			
Hemodynamics^			X	X						
Surgical data^^				X						
Postoperative data^^^										X
Clinical Frailty Scale	X									
**LABORATORY TESTS:**										
Blood samples•		X		X		X		X	X	
Cytokines +Interleukins••		X		X			X	X		
[TIMP-2 xIGFBP7], U-creatinine			X		X					
hs-TNI		X		X		X				
Arterial lactate, glc, Na	X°	X		X	X	X				
IFABP		X			X		X			
AST, ALT, PT-INR	X					X		X	X	

*Patients having open surgery, 4-6 days postoperative, closest possible weekday;
**Total dose of norepinephrine during surgery (mcg), divided by minutes of surgery and weight of patient;
^Arterial Blood Pressure, Heart Rate (before induction), Central Venous Pressure and Cardiac Index (after insertion, and thereafter every 30 min during the resection phase), capillary refill time, hourly during surgery.
^^Performed surgical procedure, Duration of surgery, total Pringle time
^^^complications (SweLiv Registry), length of stay in hospital, radicality of resection
•WBC, CRP, PLT, Albumin, Hemoglobin, p-Creatinine
••cytokines / interleukins: IL-1β IL-6, IL-8, IL-10, MCP-1, SDF-1α, ICAM, C3a, C5b-9.
°only S-Na
