# Supplementary material for: Argipressin for prevention of blood loss during liver resection: a study protocol for a randomised, placebo-controlled, double-blinded trial (ARG-01)
Source: BMJ Open. 2023 Aug 24;13(8):e073270. doi: 10.1136/bmjopen-2023-073270 (PMC10450082; doi:10.1136/bmjopen-2023-073270)
Supplement: Supplementary data [file bmjopen-2023-073270supp004.pdf]

Statistical Analysis Plan

Influence of Argipressin on blood loss during liver resection; a double-blinded, randomized placebo-controlled trial (ARG-01)

____

Kristina Tempelman Svennerholm
MD, PhD, Principal Investigator

____

Ellinor Wisén
MD, Co-Investigator

____

Aldina Pivodic
MSc, Study Statistician

February 27, 2023

Authored by:
Aldina Pivodic
aldina.pivodic@apnc.se
+46 709 47 14 12

Revisions:

Nr	Description	Date

Wisén E, *et al. BMJ Open* 2024; 13:e073270. doi: 10.1136/bmjopen-2023-073270

Table of Contents

Abbreviations....4
1 Study Objectives....5
1.1 Primary Objective....5
1.2 Secondary Objectives....5
2 Study Design....5
2.1 Treatment Groups....5
2.2 Flow Chart....5
3 Study Populations....8
3.1 Intention-to-Treat Population....8
3.2 Per Protocol Population....8
3.3 Safety Population....8
4 Study Variables....9
4.1 Baseline Variables....9
4.2 Medical History....9
4.3 Prior Medications....9
4.4 Efficacy Variables....10
4.4.1 Primary Variable....10
4.4.2 Secondary Variables....10
4.5 Safety Variables....11
4.5.1 Exposure and Compliance....11
4.5.2 Adverse Events....12
5 Statistical Methods....12
5.1 Sample Size....12
5.2 General Methodology....12
5.3 Adjustment of Type I Error....14
5.4 Patient Disposition and Datasets Analyzed....14
5.5 Protocol Deviations....14
5.6 Demographics and Baseline Characteristics....14
5.7 Medical History....14
5.8 Prior Medications....15

5.9 Efficacy Analyses 15
5.9.1 Primary Analysis 15
5.9.2 Secondary Analyses 15
5.10 Subgroup Analyses 15
5.11 Safety Analyses 16
5.11.1 Exposure and Compliance 16
5.11.2 Adverse Events 16
6 Interim Analyses 16
7 Changes from Study Protocol 17
8 Planned Tables and Figures 18

Abbreviations

**ASA**	American society of anesthesiologists
**BMI**	Body mass index
**CFS**	Clinical frailty scale
**CRP**	C-reactive protein
**CVP**	Central venous pressure
**HCC**	Hepatocellular carcinoma
**HR**	Hazard ratio
**Hs-TNI**	High-sensitivity troponin I
**I-FABP**	Intestinal fatty acid-binding protein
**IGFBP**	Insulin-like growth factor-binding protein
**IL**	Interleukin
**ITT**	Intention to treat
**MAP**	Mean arterial pressure
**MedDRA**	Medical dictionary for regulatory activities
**MCP**	Monocyte chemoattractant protein
**MMRM**	Mixed models for repeated measures
**OR**	Odds ratio
**POD**	Postoperative day
**PP**	Per protocol
**PT**	Preferred term
**RR**	Relative risk
**SDF**	Stromal cell-derived factor
**SOC**	System organ class
**TAP**	Transversus abdominis plane
**TIMP**	Tissue inhibitor of metalloproteinases
**TIVA**	Total intravenous anesthesia
**WBC**	White blood cells

Wisén E, *et al. BMJ Open* 2024; 13:e073270. doi: 10.1136/bmjopen-2023-073270

1 Study Objectives

1.1 Primary Objective

To determine if infusion of Argipressin during liver resection surgery reduces blood loss compared to placebo.

1.2 Secondary Objectives

Secondary objectives include assessment of argipressin's effects on the need for blood transfusions, as well as on the inflammatory response. Exploration of potential effects on kidney, cardiac and intestinal tissue biomarkers will be performed. The duration of the surgery- and the resection phase as well as use of PM, in addition to radicality of resection will be determined. Perioperative hemodynamics, use of vasopressor and achievement of low CVP goals will be evaluated. Finally, total length of stay in hospital and postoperative complications at 30 days are noted.

2 Study Design

This single centre, double-blinded, randomized, placebo-controlled trial is performed at Sahlgrenska University Hospital. The aims are to investigate the effect of Argipressin on blood loss, transfusion requirements and inflammatory response during liver resection. Participants will be stratified according to planned surgical technique (open or laparoscopic surgery) and planned extent of resection (“large” or ”small”) before randomization to treatment with argipressin or placebo during surgery. Markers of kidney, cardiac and intestinal damage will be evaluated during and after surgery. More extensive testing of inflammatory markers will be performed in a limited number of patients (subgroup) in each arm.

2.1 Treatment Groups

There are two randomized treatment groups in this study:

1. Argipressin
2. Placebo

2.2 Flow Chart

Overall study design is shown in the Flow chart below.

Wisén E, *et al. BMJ Open* 2024; 13:e073270. doi: 10.1136/bmjopen-2023-073270

ARG-01 Flow Diagram

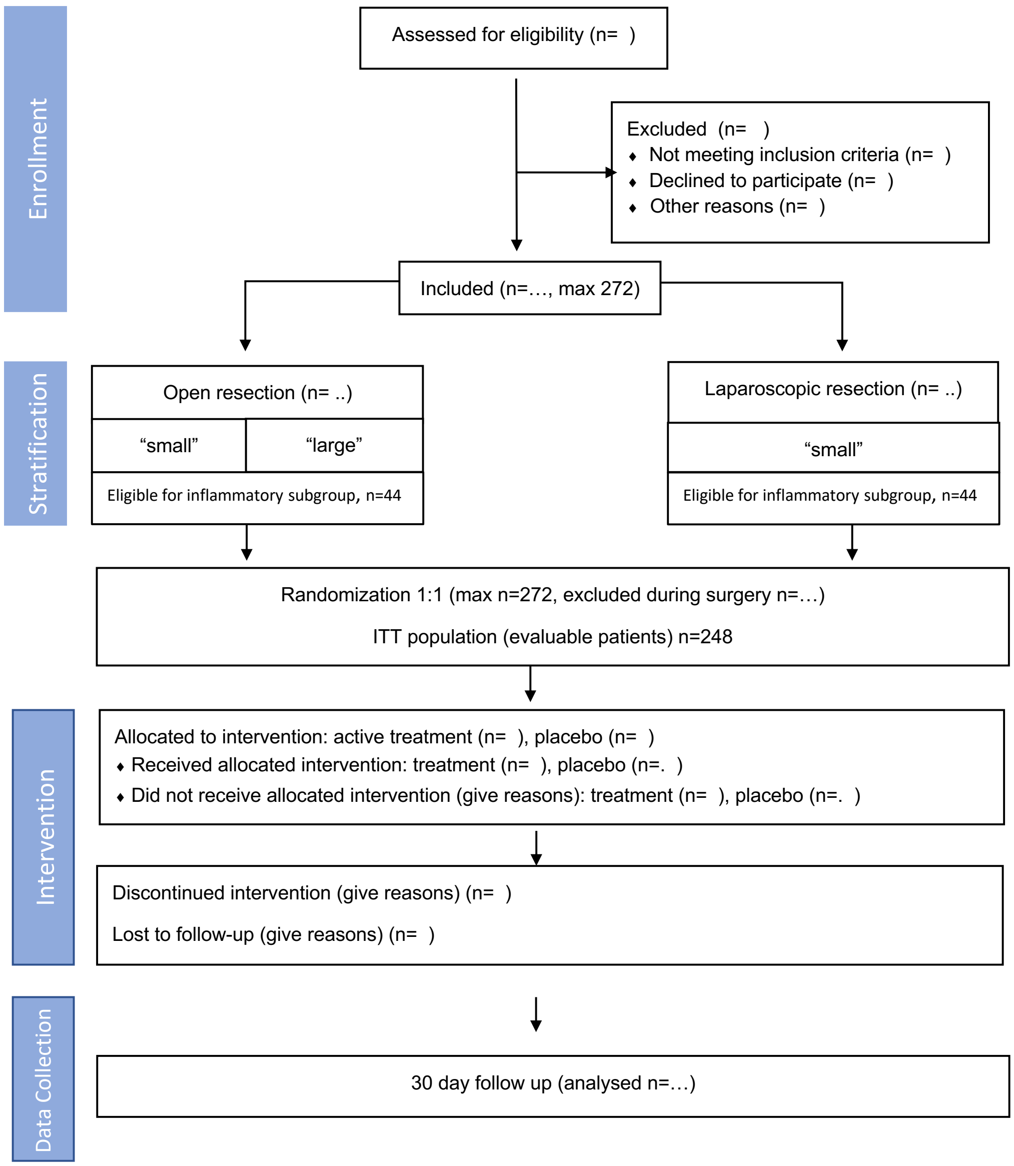

Table 1 - Overview of the study activities

	Enrolment	**Allocation**	**Intervention**			**Post operative care**				***Close -out***
**TIMEPOINT**	***Pre op visit***	**Day of surgery -** *Before anesthesia*	**Day of Surgery-** *After induction*	**Day of Surgery-** *End of surgery*	**Day of Surgery-** End of Surgery +3h	***Post op day 1*** *5 am*	***Post op day 1*** *10 am*	***Post op day 2***	***Post op day 5****	***30 day follow up***
**ENROLMENT:**										
Eligibility screen	X									
Informed consent	X									
Demographic data	X									
Allocation		X								
**INTERVENTION:**										
Study drug			**I--------------**	**-----------I**						
assessment of AEs		X	X	X	X	X	X	X	X	
**ASSESSMENTS:**										
Blood loss				X						
Blood transfusion (ml)				X			X	X	X	
Norepinephrine**				X						
Use of other inotropic drugs				X						
CVP-goal achieved				X						
Total diuresis							X			
Furosemide total dose							X			
Hemodynamics^			X	X						
Surgical data^^				X						
Postoperative data^^^										X
Clinical Frailty Scale	X									
**LABORATORY TESTS:**										
Blood samples•		X		X		X		X	X	

Wisén E, *et al. BMJ Open* 2024; 13:e073270. doi: 10.1136/bmjopen-2023-073270

Cytokines +Interleukins••		X		X			X	X		
[TIMP-2 xIGFBP7], U-creatinine			X		X					
hs-TNI		X		X		X				
Arterial lactate, glc, Na	X°	X		X	X	X				
IFABP		X			X		X			
AST, ALT, PT-INR	X					X		X	X	

*Patients having open surgery, 4-6 days postoperative, closest possible weekday;
**Total dose of norepinephrine during surgery (mcg), divided by minutes of surgery and weight of patient;
^Mean Arterial Blood Pressure, Heart Rate (before induction), Central Venous Pressure and Cardiac Index (after insertion) and thereafter every 30 min during the resection phase. Capillary refill time; hourly during surgery
^^Duration of surgery, total Pringle time
^^^complications (SweLiv Registry), length of stay in hospital, radicality of resection
•WBC, CRP, PLT, Albumin, Hemoglobin, p-Creatinine
••cytokines / interleukins: IL-1β IL-6, IL-8, IL-10, MCP-1, SDF-1α, ICAM, C3a, C5b-9.
°only S-Na

3 Study Populations

3.1 Intention-to-Treat Population

The intention-to-treat (ITT) population will include all patients that were randomized into the study. ITT analyses will be performed on as-randomized basis.

3.2 Per Protocol Population

The per-protocol (PP) population will include all patients that were randomized into the study and that do not have any major protocol violations in the study affecting the outcome. The final PP population will be defined in blinded manner, after the clean file of the study database and before the database lock and unblinding of the study treatment. PP analyses will be performed on as-treated basis.

Major protocol violations will be collected during the study and may include following but are not limited to:

- Excessive bleeding not related to resection phase, such as injury of vena cava.

3.3 Safety Population

The safety population will include all patients that were randomized into the study and that have undergone study intervention with either argipressin treatment or placebo. Safety analyses will be performed on as-treated basis.

4 Study Variables

4.1 Baseline Variables

Following baseline variables will be described:

- Patient characteristics
  - Sex (Male, Female)
  - Age (years)
  - Weight (kg)
  - Height (cm)
  - Body mass index (BMI) (kg/m^2)
  - American society of anesthesiologists (ASA) class
  - Clinical frailty scale (CFA)
- Surgery details
  - Performed surgical technique (Laparoscopic, Open)
  - Change of surgical technique (Laparoscopic to Open)
  - Robot-assisted surgery (Yes, No)
  - Performed extent of surgery (Large, Small)
  - Change of extent of surgery (Large to Small, Small to Large)
  - Indication for surgery (Colorectal metastasis, Gallbladder tumor, cholangiocarcinoma/Klatskin tumor, Hepatocellular carcinoma (HCC), Benign, Other)
- Included in substudy

4.2 Medical History

Following medical history will be described:

- Ischemic heart disease (n, %)
- Cardiac failure (n, %)
- Hypertension (n, %)
- Diabetes mellitus type I (n, %)
- Diabetes mellitus type II (n, %)
- Systemic inflammatory disease (n, %)
- Any of the above (n, %)

4.3 Prior Medications

Following medications will be described:

- B01 Antithrombotic agents (n, %)

Wisén E, *et al. BMJ Open* 2024; 13:e073270. doi: 10.1136/bmjopen-2023-073270

- C01 Cardiac therapy (n, %)
- C02 Antihypertensives (n, %)
- C03 Diuretics (n, %)
- C07 Beta blocking agents (n, %)
- C08 Calcium channel blockers (n, %)
- C09 Agents acting on the renin-angiotensin system (n, %)
- H02 Corticosteroids for systemic use (n, %)
- L Antineoplastic and immunomodulation agents (last 2 weeks) (n, %)
- Any of above (n, %)

4.4 Efficacy Variables

4.4.1 Primary Variable

This study's primary variable is:

- Blood loss (ml) at the end of surgery, measured according to the investigator's instructions, by visual assessment of suction devices and gauze, and subtraction of ascites and irrigation fluids.

4.4.2 Secondary Variables

This study's secondary variables aimed to be confirmed are the following:

- The proportion of patients receiving blood transfusions and the volume of blood transfusions (mL) during surgery and hospitalization (but not longer than postoperative day (POD) 2 for laparoscopic and POD 5 for open surgery).
- Duration of Pringle's manoeuvre (PM), resection phase and surgery (min).
- Length of stay in hospital (days).
- Change of levels of plasma creatinine from baseline to POD 1, 2 and 5. Urine creatinine and urine [Tissue inhibitor of metalloproteinases (TIMP)-2] x [Insulin-like growth factor-binding protein (IGFBP)-7] (Nephrocheck®) from baseline to 3 h after end of surgery.
- Change of plasma high-sensitivity troponin I (hs-TnI) levels from baseline to end of surgery and POD 1.
- Change of plasma lactate and intestinal fatty acid-binding protein (I-FABP) levels of from baseline to end of surgery, at 3 h after surgery and POD 1.
- Inflammatory response: change in levels of white blood cells (WBC), C-reactive protein (CRP), platelets and albumin, Interleukin (IL)-1β IL-6, IL-8, IL-10, Monocyte chemoattractant protein (MCP)-1, Stromal cell-derived factor (SDF)-1α, ICAM, C3a, C5b-9 from baseline, to end of surgery and POD 1 and 2.
- Total dose of noradrenaline during surgery, achievement of CVP goal, signs of digital hypoperfusion, total urine output and total dose of furosemide at POD 1.
- Postoperative complications documented at POD 30, n (%), any and number of the following complications:

    - Liver failure
    - Renal failure
    - Bile leakage
    - Ascites
    - Bleeding
    - Vessel complications liver/gallducts
    - Biliary stricture
    - Gastrointestinal perforation/leakage
    - Other gastrointestinal complication
    - Abdominal wall complication
- Radicality of resection.

4.5 Safety Variables

4.5.1 Exposure and Compliance

As study treatment will be administered intravenously by hospital staff during surgery, and all samples will be obtained during hospitalization in conjunction with surgery, compliance is expected to be 100%.

Following surgery details and information about study drugs will be described:

- Surgery details
    - Excessive blood loss due to unexpected complication (Yes, No)
    - Use of other inotropic drugs (Yes, No)
    - Anesthesia method (Fentanyl + Sevoflurane, Remifentanil + Sevoflurane, Total intravenous anesthesia (TIVA) propofol + Remifentanil, Other)
    - Regional anesthesia (Epidural, Intrathecal morphine, Transversus abdominis plane (TAP), Rectus sheath block, None)
    - Betapred received during surgery (Yes, No)
- Study drug
    - Infusion duration (min)
    - Administered volume of study drug (mL)
    - Was the study drug prematurely discontinued (Yes, No)
    - Reason for discontinuation (Hypersensitivity to study drug or any of the excipients, Unexpected serious event jeopardizing the safety of the patient, Other)
    - Randomization code broken (Yes, No)
- Study duration (days)

Wisén E, *et al. BMJ Open* 2024; 13:e073270. doi: 10.1136/bmjopen-2023-073270

4.5.2 Adverse Events

All adverse events (AE) from the first visit onwards will be collected in the study. Treatment emergent AEs are those that have started on or after the first dose of study drug provided in the study, and will be summarized. AEs occurring prior to the provided study intervention will also be tabulated. The AEs will be collected continuously during the study and will be coded using Medical dictionary for regulatory activities (MedDRA) to standardized system organ class (SOC) and preferred term (PT) before summarized.

5 Statistical Methods

5.1 Sample Size

Calculation of sample size was performed based on data from open and laparoscopic liver resections performed at Sahlgrenska University Hospital during the period Jan 2019-Oct 2020. When applying log-transformation on the blood volume variable normal distribution was obtained. The sample size calculation was performed using two-sided t-test on the log-transformed variable that will correspond to the selected method for the primary analysis, i.e. generalized linear model with lognormal distribution. To be able to find at least 35% reduction in the mean blood loss in the treatment arm compared to the placebo arm (962 ml vs 625 ml, respectively), i.e. a difference of at least 0.43 in the log-scale assuming SD of 1.17, with a level of significance of 5%, and a power of 80%, 118 patients are needed to be included in each arm. Assuming a dropout rate of 5%, 248 patients in total will need to be included, 124 patients in each arm. A limited number of patients will be discovered fulfilling the exclusion criteria after the randomization and first after they have started the surgery. Since the investigational product is given from the start of the surgery these patients will be part of the Safety population and their safety outcome regarding 30-day survival will be collected. Their surgery will be immediately stopped, and they will be excluded from the study and the ITT population from all efficacy analyses. To account for these dropouts 248 ITT evaluable patients will be included, but not more than 272 randomized patients in total.

For the subgroup analysis 44 patients in the argipressin and placebo group respectively, will be included (total n= 88). The number of patients selected for extended inflammatory testing is based on a pilot study (unpublished data) performed at Sahlgrenska University Hospital.

5.2 General Methodology

All analyses will be performed using SAS software version 9.4 or later (SAS Institute Inc., Cary, NC, USA).

Descriptively, continuous variables will be described by mean, SD, median, interquartile range, and range as appropriate, and categorical variables by number and percentage.

For test between two groups Fisher's exact test will be used for dichotomous variables, Mantel-Haenszel Chi-square trend test for ordered categorical variables, Chi-square test for non-ordered categorical variables, and two-sample t-test or Mann-Whitney U-test for continuous variables as appropriate based on their distribution.

Analysis of normally distributed outcome variables will be performed using general linear model adjusted for randomization strata (laparoscopic, open small and open large surgeries), and those that are lognormally distributed generalized linear model will be used with lognormal distribution with the same adjustment. In case of not fulfilling model assumptions non-parametric unadjusted Mann-Whitney U-test will be used for continuous outcome variables. Dichotomous variables will be analysed using binary logistic regression adjusted for randomization strata as above. Number of complications per follow-up time will be analysed using Poisson regression including adjustment for randomization strata. Time-to-event data, such as length of stay in hospital, will be graphically described using Kaplan-Meier technique and analysed using Cox regression model, with the same adjustment. Assumption of proportional hazard will be checked by studying interactions with time in study. From these analyses, difference in least square means, relative risk (RR), odds-ratio (OR), and hazard ratio (HR) with 95% CI between the treatment groups and associated p-values will be the main measures presented.

The secondary variables that will be included in the confirmatory fixed sequential testing per the order below following the confirmation of the primary analysis, with 0.05 significance level, will be:

- The volume of blood transfusions during surgery and hospitalization
- The proportion of patients receiving blood transfusion

All other secondary endpoints will be evaluated in exploratory manner, and hence not adjusted for multiplicity. All exploratory tests will be cautiously considered, considering the magnitude and clinical relevance of the results.

Tests for interleukin and cytokine levels (IL-1b IL-6, IL-8, IL-10, MCP-1, SDF-1a, ICAM, C3a, C5b-9) will only be analysed in a subgroup of 44 patients in each parallel group (total n=88). The results will be presented as explorative data.

No interim analysis is planned. Data will be analyzed by the statistician before being presented to the research group.

Missing data for the confirmatory analysis is not expected. Missing data of variables with repeated measures will not be imputed but handled as missing completely at random in the MMRM.

All primary and secondary variables will be analyzed both for the ITT (main analysis) and PP (robustness analysis) population.

5.3 Adjustment of Type I Error

Fixed sequential testing will be applied with alpha 0.05 for the confirmatory analysis specified in General Methodology above.

5.4 Patient Disposition and Datasets Analyzed

The number of patients included in each of the ITT, PP and safety populations will be summarized for both treatment groups. Patients who completed the study and those who withdrew from study prematurely will also be presented with a breakdown of the reasons for withdrawal by treatment group for the ITT, PP, and safety populations.
Following reasons for discontinuations regarding interruption of study medication will be captured in the study:

- Patient decision
- Safety concerns
- Incorrect enrollment
- Perioperative change of surgical procedure
- Other

Following reasons for discontinuation regarding 30-day follow-up will be captured in the study:

- Withdrawn consent
- Death
- Lost to follow-up
- Other

5.5 Protocol Deviations

Major protocol deviations are those that are considered to have an impact on the study outcome. The number of patients with major protocol deviations will be summarized per treatment group, and listed.

5.6 Demographics and Baseline Characteristics

Demographics and baseline characteristics will be summarized by treatment group for the ITT and PP population and analyzed according to the methods described in section General Methodology above.

5.7 Medical History

Medical history will be summarized for each treatment group for the ITT population.

5.8 Prior Medications

Prior medications will be summarized for each treatment group for the ITT population.

5.9 Efficacy Analyses

5.9.1 Primary Analysis

The primary endpoint, blood loss at the end of surgery, will be evaluated on the ITT population comparing all patients treated with argipressin vs. placebo, on 0.05 significance level. The primary analysis will be performed using a generalized linear model with lognormal distribution, adjusted for randomization strata of laparoscopic, open small, and open large surgery. Relative risk (RR) with 95% CI and p-value will be described. Diagnostic plots will be reviewed to assure that the model assumptions are fulfilled. Graphically, the primary endpoint will be described using boxplots.

Analysis performed on the PP population will serve as robustness analysis.

5.9.2 Secondary Analyses

The secondary analyses will be performed as defined in the General Methodology above on the ITT and the PP population. Actual values and change from baseline will be described for study visits. All raw data for continuous variables over time will be shown as boxplots per treatment group.

5.10 Subgroup Analyses

The effect of treatment on the primary endpoint, blood loss, and on the need for transfusion will be studied for following subgroups:

- Performed surgical technique (Open vs Laparoscopic)
- Performed extent of surgery (Small vs Large)
- Performed extent of surgery within open surgery (Small vs Large)
- Indication for surgery (divided into 1-2 largest categories vs other)
- Hepatic cirrhosis (yes vs no)
- Age (≤median vs >median)

The final list of subgroups might be updated during the study before the database lock, unblinding and programming of the study database and analyses.

The analyses will include investigation of the interaction between the intervention and the considered variable using the same regression methods as specified above for blood loss and need for transfusion. The results will be graphically described in forest plots.

5.11 Safety Analyses

5.11.1 Exposure and Compliance

Exposure, compliance, and surgery related variables will be summarized for each treatment group. The summaries will be provided for the safety population.

5.11.2 Adverse Events

Treatment-emergent AEs will be included in the summaries for the safety population. A summary of subjects reporting at least one of the following AEs will be presented in an overview table:

- Any AE
- Any SAE
- Any treatment-related AE (possible, probable or definite will be considered related)
- Any treatment-related SAE (possible, probable or definite will be considered related)
- Any procedure-related AE (possible, probable or definite will be considered related)
- Any procedure-related SAE (possible, probable or definite will be considered related)
- Any AE leading to discontinuation
- Death

Summaries per CTCAE (v5) presenting n (%) of AEs and n (%) of subjects with at least one AE will be provided for:

- All AEs (includes all serious and non-serious AEs)
- All AEs by maximum reported intensity (mild, moderate, severe)
- All AEs by causality
- All SAEs
- All AEs leading to discontinuation

AEs collected prior to first study dose provided will be separately tabulated.

6 Interim Analyses

None.

7 Changes from Study Protocol

This SAP is based on the study protocol version 3

Wisén E, *et al. BMJ Open* 2024; 13:e073270. doi: 10.1136/bmjopen-2023-073270

8 Planned Tables and Figures

The number and order of the tables and figures might change as appropriate.

Table Number	Table Title
**Table 1.1**	Patient disposition and data sets analyzed (ITT population)
**Table 1.2**	Protocol violation leading to exclusion from PP population (ITT population)
**Table 2.1**	Demographics and baseline characteristics (ITT population)
**Table 2.2**	Demographics and baseline characteristics (PP population)
**Table 3.1**	Medical history (ITT population)
**Table 3.2**	Prior medications (ITT population)
**Table 4.1**	Confirmatory analyses of the primary and secondary efficacy variables (ITT population)
**Table 4.2**	Confirmatory analyses of the primary and secondary efficacy variables (PP population)
**Table 5.x.1**	Exploratory analyses of the secondary efficacy variables XXXX (ITT population)
**Table 5.x.2**	Exploratory analyses of the secondary efficacy variables XXXX (PP population)
**Table 6.1**	Subgroup analyses of the primary and secondary variables (ITT population)
**Table 6.2**	Subgroup analyses of the primary and secondary variables (PP population)
**Table 7**	Exposure, compliance and surgery data (Safety population)
**Table 8.1**	Summary of adverse events (Safety population)
**Table 8.2**	Adverse events by SOC and PT (Safety population)
**Table 8.3**	Adverse events by SOC and PT and maximum severity (Safety population)
**Table 8.4**	Adverse events by SOC and PT and causality assessment (Safety population)
**Table 8.5**	Serious adverse events by SOC and PT (Safety population)
**Table 8.6**	Adverse events leading to discontinuation by SOC and PT (Safety population)
**Table 8.7**	Adverse events collected prior to first dose of investigational product (Safety population)

Figure Number	Figure Title
**Figure 1.x**	Boxplots for XXXX per treatment group (ITT population)
**Figure 2.**	Kaplan-Meier for time to discharge from hospital per treatment group (ITT population)
**Figure 3.**	Forest plot for subgroup analyses of primary and secondary variables (ITT population)

Wisén E, *et al. BMJ Open* 2024; 13:e073270. doi: 10.1136/bmjopen-2023-073270
